# Modulating the sense of agency in functional neurological disorder using real-time fMRI neurofeedback: a proof-of-concept study^☆^

**DOI:** 10.1016/j.nicl.2025.103899

**Published:** 2025-11-02

**Authors:** Eliane Müller, Serafeim Loukas, Salome Häuselmann, Cristina Concetti, Dimitri Van De Ville, Nicolas Gninenko, Selma Aybek

**Affiliations:** a Department of Neurology, Faculty of Science and Medicine, University of Fribourg, Switzerland; b Neuro-X Institute, Ecole Polytechnique Fédérale de Lausanne (EPFL), Geneva, Switzerland; c Department of Neurology, Psychosomatic Medicine Unit, Inselspital Bern University Hospital, Switzerland; d Graduate School of Cellular and Biomedical Sciences (GCB), University of Bern, Switzerland; e Department of Radiology and Medical Informatics, University of Geneva, Switzerland

**Keywords:** Functional MRI, Neurofeedback, Functional neurological disorder, Sense of agency, Temporoparietal junction

## Abstract

**Background:**

Disrupted sense of agency (SoA), the perceived loss of control over one’s actions, is common in functional neurological disorder (FND) and linked to abnormal activation in agency-related networks, particularly the right temporoparietal junction (rTPJ). This proof-of-concept study tested whether fMRI neurofeedback (NF) could enhance SoA in FND by targeting the rTPJ.

**Methods:**

In this single-arm feasibility study, 18 patients with mixed FND symptoms completed three NF trainings during an adaptive visuomotor task, receiving intermittent visual feedback of their rTPJ activity. Pre- and post-training assessments included subjective agency ratings, neuroimaging during a transfer task, and clinical evaluations.

**Results:**

Group-level subjective agency significantly increased after training (*p* = 0.0083), driven by responders (n = 8) who exhibited rTPJ (*p* = 0.042) and supplementary motor area (SMA) upregulation (*p* = 0.015) in the first session, alongside consistent task engagement. Behavioral gains coincided with trend-level decreases in rTPJ–dorsolateral prefrontal cortex (dlPFC) connectivity (*p* = 0.098), while non-responders showed reduced rTPJ–SMA connectivity (*p* = 0.008). No group-level clinical improvements were observed, but higher initial rTPJ (*p* = 0.03, ρ = –0.78) and dlPFC (*p* = 0.03, ρ = –0.75) activation were negatively associated with changes in self-reported core symptoms.

**Conclusion:**

NF from rTPJ activity is feasible in FND and may enhance explicit SoA in a subset of patients through early neural modulation and sustained engagement. High inter-individual variability underscores the need for sham-controlled trials with optimized, engaging feedback designs and longitudinal monitoring to identify patients who could benefit from NF.

## Highlights

• Functional MRI neurofeedback can enhance explicit sense of agency in FND patients.
• Responders showed early activation of the agency network during training.
• Responders and non-responders showed distinct functional connectivity changes.
• Increased explicit agency may heighten symptom awareness before clinical gains.

## 1 Introduction

Functional neurological disorder (FND) is a common and complex condition that presents with a variety of neurological symptoms, such as motor dysfunction (e.g., tremor, limb weakness, paralysis, or gait disorder), sensory disturbances (e.g., tingling, numbness, or pain), and functional seizures (Espay et al., 2018). A core clinical feature of FND, observed across symptom domains, is a subjective lack of control over one’s movements; i.e., a disrupted sense of agency (SoA), despite evidence of preserved motor pathways (Hallett, 2010). This altered SoA in FND has been demonstrated using various experimental paradigms targeting either *implicit* or *explicit* components of agency. Implicit agency refers to the pre-reflective, sensorimotor feeling of control over one’s actions, while explicit agency involves the conscious attribution or judgment of authorship over an action (Synofzik et al., 2008). Implicit agency is typically assessed using paradigms such as action-effect binding (Kranick et al., 2013) and sensory attenuation (Macerollo et al., 2015, Pareés et al., 2014), which rely on automatic processes. In contrast, explicit agency is measured through tasks such as visuomotor manipulation (Bühler et al., 2024, Stager et al., 2022) or virtual reality-based paradigms (Nahab et al., 2017), where participants are asked to consciously judge the degree of agency they experienced during the task.

Converging evidence from neuroimaging research indicates that the SoA is supported by a network of functionally interconnected brain regions. These regions include the left and right temporoparietal junctions (lTPJ and rTPJ), the bilateral dorsolateral prefrontal cortex (dlPFC), the supplementary motor area (SMA), the insular cortex (IC), and the cerebellum (Crivelli and Balconi, 2017, David et al., 2008, Miele et al., 2011, Seghezzi et al., 2019). Several functional magnetic resonance imaging (fMRI) studies in FND have reported disrupted functional connectivity between the rTPJ, cerebellar vermis, bilateral SMA, right IC, and sensorimotor cortex (Baek et al., 2017, Maurer et al., 2016, Voon et al., 2010), as well as dysfunction involving the right dlPFC, pre-SMA, and the rTPJ (Bühler et al., 2024, Nahab et al., 2017, Voon et al., 2010). Among these affected brain regions, the rTPJ has emerged as a critical hub for agency processing, responsible for comparing predicted sensory consequences of movement with the actual sensory feedback (Farrer et al., 2008). In FND, hypoactivity of the rTPJ has been associated with impaired movement control and a diminished SoA compared to healthy individuals (Bühler et al., 2024, Voon et al., 2010), highlighting the rTPJ as a promising target for neuromodulatory interventions.

Non-invasive brain stimulation techniques, such as transcranial magnetic stimulation (TMS), have already been explored as a way of modulating the SoA by targeting the rTPJ (Khalighinejad and Haggard, 2015). In healthy individuals, TMS-induced inhibition of the posterior parietal cortex (overlapping with the rTPJ) has been shown to decrease the SoA (Preston and Newport, 2008, Ritterband-Rosenbaum et al., 2014, Zito et al., 2020). However, a recent TMS study in FND patients showed that while excitatory stimulation of the rTPJ effectively modulated its brain activity compared to sham stimulation, this modulation did not lead to measurable improvements in explicit agency (Bühler et al., 2024).

Neurofeedback (NF) provides an alternative approach for modulating brain activity, enabling individuals to learn self-regulation through real-time visual or auditory feedback from targeted brain regions (Sitaram et al., 2017, Thibault et al., 2018, Weiskopf et al., 2007). A recent electroencephalography (EEG) NF study targeting agency showed that healthy individuals could successfully modulate central theta power during training, which was associated with improved visuomotor performance. However, this did not result in measurable changes in their explicit SoA (Zito et al., 2023).

While EEG NF offers high temporal resolution, it suffers from limited spatial resolution and specificity (Weiskopf et al., 2007). In contrast, fMRI NF, though slower, offers superior spatial resolution and can more precisely target dysfunctional brain regions (Sulzer et al., 2013). Technological advances over the last two decades have improved the precision, real-time processing, and usability of fMRI NF, expanding its research applications across a range of neurological and psychiatric disorders (Sulzer et al., 2013). Promising results have been reported from early-stage clinical studies, including in Parkinson’s disease (Subramanian et al., 2016), stroke (Liew et al., 2016), phobias and post-traumatic stress disorder (PTSD; (Askovic et al., 2023, Koizumi et al., 2016, Reiter et al., 2016)), depression (Koush et al., 2017b, Linden et al., 2012, Mathiak and Keller, 2021, Pereira et al., 2022), and tinnitus (Gninenko et al., 2024, Emmert et al., 2017, Haller et al., 2010). Although fMRI NF of the rTPJ has been shown to be feasible in both healthy and clinical populations (Kruse et al., 2024, Saxena et al., 2023), it has not yet been applied in FND to directly target disrupted agency processing.

This single-arm proof-of-concept study investigated whether individuals with FND can learn to upregulate activity in the rTPJ, a key hub in the SoA network, using activity-based fMRI NF. Participants completed a five-visit protocol, comprising three NF training sessions and pre- and post-intervention clinical assessments. The primary objective was to assess the feasibility and tolerability of implementing this protocol in a clinical sample of patients with FND. Secondary objectives were to evaluate whether fMRI NF would (i) increase the subjective SoA as measured behaviorally, (ii) enhance activation in the rTPJ and related network regions, (iii) modulate task-related functional connectivity, and (iv) improve clinician-rated and self-reported symptom severity, and quality of life. We hypothesized that fMRI NF training would increase rTPJ activation and support functional restoration within the agency network, with these neural changes contributing to improvements in the agency experience and symptom burden.

## 2 Methods

### 2.1 Participants

Twenty-one FND patients with motor (F44.4), functional seizure (F44.5), or mixed symptom type (F44.7) were recruited from the University Hospital in Bern (Inselspital) and the Cantonal Hospital in Fribourg (HFR), Switzerland. The diagnosis was confirmed according to the ICD-10 and DSM-5 (DSM5, 2013) criteria and positive signs (Stone and Carson, 2015) by a board-certified neurologist. Participants were excluded if they met any of the following conditions: major neurological comorbidities, contraindications to MRI, substance or alcohol abuse, current severe psychiatric condition (acute suicidality, psychosis), pregnancy, breastfeeding, or being younger than 16 years old. All participants provided written informed consent. The study received approval from the local ethics committee of the Canton of Bern, Switzerland (SNCTP000004529), and was conducted in accordance with the principles of the Declaration of Helsinki. The study protocol was preregistered on ClinicalTrials.gov (NCT05086380). Participants were compensated monetarily for their time, in addition to receiving reimbursements for travel expenses.

### 2.2 Study design and procedure

**Fig. 1:**
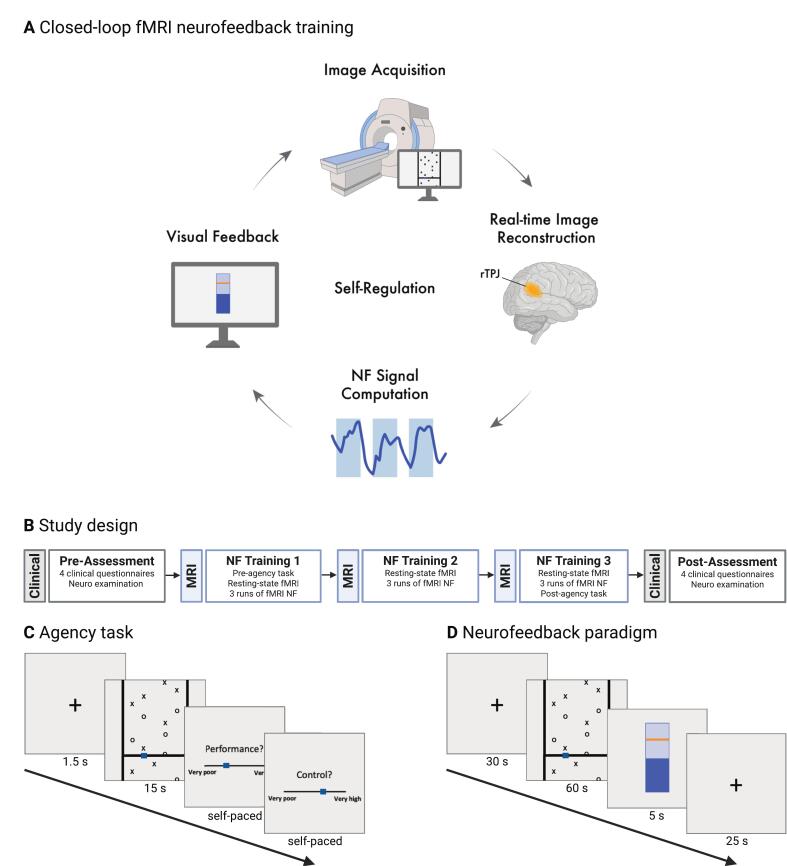
Experimental protocol and fMRI paradigm. A) Conceptual overview of the real-time fMRI neurofeedback (NF) training targeting the right temporoparietal junction (rTPJ) to modulate the sense of agency. B) Study design of the single-arm, proof-of-concept NF trial consisting of five visits, approximately one week apart. Before the clinical pre-assessment, participants were screened for eligibility and provided informed consent. C) The agency task was administered both pre- and post-NF training to assess behavioral and neural changes. Participants used a cursor to catch falling targets (X’s) while avoiding distractors (O’s), then rated their perceived control (judgement of agency) and performance (judgement of performance). In half of the trials, control over the cursor was disrupted (*turbulence*), while in the other half, control was maintained (*non-turbulence*). D) During NF training, participants performed a modified agency task. Instead of providing subjective ratings, they received intermittent visual feedback in the form of a blue bar reflecting average rTPJ activity. The game conditions (*turbulence* vs. *non-turbulence*) were dynamically adapted based on rTPJ activation. Created in BioRender (https://BioRender.com/ky3v97k). (For interpretation of the references to colour in this figure legend, the reader is referred to the web version of this article.)

This single-arm feasibility study consisted of five separate visits, including pre- and post-clinical assessments, as well as three neuroimaging sessions with fMRI NF training (Fig. 1, Fig. 1). Visits were scheduled approximately one week apart, resulting in a total study duration of around four weeks (mean 30.1 ± 8.1 days).

During the first visit, participants underwent an eligibility screening and a clinical evaluation, which included a neurological examination. They also completed questionnaires assessing their emotional state, quality of life, and subjective symptom severity (see section 2.3 for details).

During the second visit, participants performed the pre-intervention agency task (pre-agency) during fMRI to establish a baseline of brain activity, followed by a resting-state sequence and their first fMRI NF training session.

During visit three, participants again underwent a resting-state scan before the second NF training session.

During visit four, the final resting-state sequence and fMRI NF training session were performed, followed by the post-intervention agency task (post-agency). After each neuroimaging session, participants reported the cognitive strategies they had used to regulate the rTPJ and rated changes in mood, motivation, and subjective symptom severity.

During visit five, participants underwent a final clinical evaluation, including a neurological examination and follow-up questionnaires.

The study was reported in accordance with the *Consensus on the Reporting and Experimental Design of Clinical and Cognitive-Behavioral Neurofeedback Studies (CRED-nf)* checklist (Ros et al., 2020); see Supplementary Materials).

### 2.3 Clinical assessments

During the first and final study visits, participants underwent clinical assessments to evaluate symptom severity, mood, and the impact of symptoms on the quality of life. Clinician-rated symptom severity was assessed using the Clinical Global Impression (CGI) scale (Busner and Targum, 2007) (0 = no symptoms to 7 = among the most severely ill patients) and the Simplified Version of the Functional Movement Disorder Rating Scale (S-FMDRS) (Nielsen et al., 2017). Positive neurological signs and the degree of motor weakness were also recorded (Stone and Carson, 2015). Participants rated their subjective symptom severity (SSS) of their core FND symptoms on a visual analogue scale (VAS) ranging from 0 (no symptoms) to 100 (the most severe symptoms experienced). Illness duration was calculated as the number of years between symptom onset and the date of study inclusion. Current use of psychotropic medication, such as opioids, antidepressants, neuroleptics, benzodiazepines, and antiepileptics, was documented. Mood was evaluated using the Spielberger State-Trait Anxiety Inventory (STAI) (Spielberger, 2010) and the Beck Depression Inventory (BDI) (Beck et al., 1961). The impact of functional symptoms on quality of life was assessed using the Quality of Life in Neurological Disorders (Neuro-QoL) questionnaire (Cella et al., 2012) and the 36-item Short Form Health Survey (SF-36) (Ware and Sherbourne, 1992).

### 2.4 Neuroimaging data acquisition

MRI data were collected on a 3 T Siemens PRISMA scanner with a 64-channel head and neck coil at the Human Neuroscience Platform (HNP) of the Campus Biotech, in Geneva, Switzerland. Further details on the acquisition sequences are provided in Supplementary Materials A.

#### 2.4.1 Agency task

The behavioral task used during the pre- and post-agency assessments was adapted from Metcalfe and Greene (Metcalfe and Greene, 2007) and has previously been used to investigate the SoA in both healthy controls (Cosentino et al., 2011, Miele et al., 2011, Zito et al., 2023, 2020) and patients with FND (Bühler et al., 2024, Stager et al., 2022). Each trial began with a 1.5-second fixation cross, followed by a 15-second game phase, in which participants were required to move a cursor along a horizontal bar to catch descending targets (X’s) while avoiding distractors (O’s) (see Fig. 1C). After the game phase, participants rated their perceived control over the game (judgment of agency, JoA) and their perceived performance (judgment of performance, JoP) on a 5-point Likert scale, ranging from −5 (very poor) to + 5 (very high). Unbeknownst to the participants, trial conditions were randomly alternated between *non-turbulence*, where cursor movement precisely matched button presses, and *turbulence*, in which random noise was added to 40 % of inputs to disrupt control. Each of the two conditions was presented 11 times, for a total of 22 trials (∼ 10 min).

#### 2.4.2 Real-time fMRI neurofeedback

Functional MRI NF training was conducted during study visits 2–4, using a modified version of the agency task (see section 2.4.1). This study included active NF for all participants. It did not include a sham control condition, primarily due to limited resources and the proof-of-concept nature of the study, which aimed to establish feasibility and gather initial efficacy data. Each regulation phase (game phase) lasted 60 s, followed by a 5‑second feedback display and a 25‑second rest period with a fixation cross (Fig. 1D). One run consisted of six 60‑second regulation phases, resulting in a total run duration of 9.5 min. Each session consisted of three runs, separated by longer breaks, yielding approximately 30 min of training per session. Feedback was presented as a blue thermometer bar reflecting the percent signal change (PSC) in the rTPJ during the preceding regulation period. In addition to the explicit, intermittent visual feedback, the neurofeedback task incorporated a dynamically adjusting component, classifying it as an adaptive neurofeedback paradigm, where task parameters change in real time based on neural activity (Sitaram et al., 2017). Specifically, the level of difficulty in the task switched dynamically between *turbulence* (limited cursor control) and *non-turbulence* (full control) conditions based on rTPJ activity. Higher rTPJ activity resulted in reduced task difficulty, providing implicit reinforcement and encouraging neural upregulation. Each trial began in *turbulence* mode and switched to *non-turbulence* only if the activity threshold was reached. Consequently, the time participants spent in each condition varied across trials; for example, those who struggled to upregulate rTPJ activity often spent most of the regulation phase in *turbulence*.

All participants received standardized instructions for the NF training. They were instructed to remain still, focus on the task, aim to maximize their game performance (i.e., hitting targets and avoiding distractors), and at the same time raise the blue feedback bar as close as possible to the orange reference line (which indicated maximum feedback). Additionally, participants were encouraged to explore cognitive strategies while playing the game to find effective ways to modulate the feedback signal. After each session, participants reported the cognitive strategies they had used to regulate the rTPJ, completed a custom questionnaire about their neurofeedback experience, and rated changes in mood and subjective symptom severity.

Real-time processing of the NF training data, feedback calculation, and display were implemented using the open-source software OpenNFT (Koush et al., 2017a). The activity-based feedback was computed from a single region of interest (ROI) in the rTPJ, defined from an earlier study that employed the same agency task with healthy participants (Zito et al., 2020). Specifically, the ROI corresponded to a significant activation cluster in the *turbulence* > *non-turbulence* contrast in the rTPJ, comprising 672 voxels. As pilot testing showed that the agency task did not reliably elicit rTPJ activation in individual FND patients, this group-level ROI from healthy participants by Zito et al., 2020 was used for the NF training of all participants. The blood oxygen level-dependent (BOLD) signal from this ROI during each regulation block was compared to the preceding baseline period to calculate the PSC. To optimize feedback sensitivity, OpenNFT applied adaptive scaling by dynamically adjusting the feedback range based on the average of the top and bottom 5 % activity time points of the data acquired so far (Davydov et al., 2022, Koush et al., 2017a). The threshold for switching between game conditions (*turbulence* vs. *non-turbulence*) was set at 50 % of the dynamic signal range.

### 2.5 Statistical analysis

Statistical analyses of clinical and behavioral data were conducted using Python (version 3.12.8) and MATLAB (version R2022a; The MathWorks Inc., Natick, MA, USA). Neuroimaging analyses were performed using SPM12 (Wellcome Centre for Human Neuroimaging, UCL, London; https://www.fil.ion.ucl.ac.uk/spm/software/spm12/), MATLAB, and Python.

#### 2.5.1 Clinical data and characteristics

The normality of the clinical data distributions was assessed using the Shapiro-Wilk test. For within-group comparisons (pre- to post-NF), paired *t*-tests were used for normally distributed variables, and Wilcoxon signed-rank tests for non-normally distributed data. Between-group comparisons (responders vs. non-responders) at baseline were conducted using independent *t*-tests or the Mann–Whitney *U* test, depending on the distribution of the data. Questionnaires with subscores were adjusted for multiple comparisons using the false discovery rate (FDR) at *q*_FDR_ = 0.05. For the SF-36, physical and mental component scores were computed (Ware et al., 1993). Categorical variables were analyzed using Fisher’s exact test.

#### 2.5.2 Behavioral data and definition of responders

For the JoA and the JoP ratings of the agency task, mean scores were calculated separately for the *turbulence* and *non-turbulence* conditions. As these ratings were ordinal (Likert scale), differences between conditions were analyzed using the non-parametric Wilcoxon signed-rank test. Pre- to post-NF changes in JoA were assessed using two-tailed paired *t*-tests. Responder classification was based on individual effect sizes (Cohen’s *d*) for JoA changes (*turbulence* vs. *non-turbulence*) between pre- and post-NF (see Supplementary Materials F). Participants with a large effect size (*d* > 0.8) were classified as responders, reflecting a substantial improvement in their ability to distinguish between states of control and lack of control (i.e., in their explicit SoA). Task performance, for both the agency task and the NF training task, was defined as the difference between the percentage of targets hit and distractors hit. Details on the analysis of the cognitive strategies used during NF regulation can be found in Supplementary Materials B.

#### 2.5.3 Functional MRI data analysis

Details on fMRI offline preprocessing are provided in Supplementary Materials C. A detailed description of the resting-state statistical analysis can be found in Supplementary Materials D.

##### 2.5.3.1 Agency task

For each participant, functional data from the agency task collected during study visits 2 and 4 were co-registered to the anatomical scan acquired during the initial neuroimaging session at visit 2. At the first level, a general linear model (GLM) was specified for each participant, including regressors for the game conditions (*turbulence* and *non-turbulence*), the judgement phases (JoA and JoP), and movement outliers identified in the realignment step (framewise displacement (FD) > 0.5 mm), modelled as a block design. At the second (group) level, contrast images for the *turbulence* > *non-turbulence* comparison were used to assess whole-brain activity changes between pre- and post-NF (i.e., visits 2 and 4). As a validity check, the same contrast was analyzed across both sessions combined to verify expected activation within the SoA network. Second-level models included the following covariates of no interest: age, sex, and a composite mood score derived by averaging *z*-scored depression (BDI) and trait anxiety (STAI-T) scores, due to their high intercorrelation (Spearman’s ρ > 0.8).

**Fig. 2:**
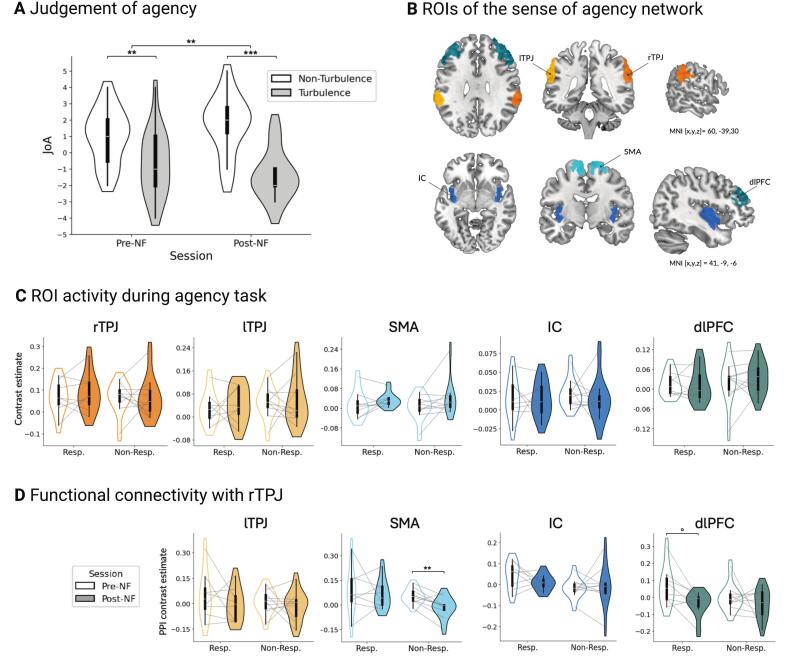
Agency task behavioral, ROI activity, and functional connectivity results. A) Group-level judgments of agency (JoA) across all participants (n = 18), comparing subjective ratings between *non-turbulence* and *turbulence* conditions before and after NF training. A significant condition effect was found at both time points (pre-NF: *p* = 0.0017; post-NF: *p* = 0.0008), with this difference between conditions significantly increasing from pre- to post-training (*p* = 0.0083). Participants showing a large increase (Cohen’s *d* ≥ 0.8) were classified as responders (n = 8), others as non-responders (n = 10). B) Sense of agency network ROIs displayed on a template brain, defined using the NF mask (TPJ) and the Glasser atlas (IC, SMA, dlPFC). C) Violin plots showing pre- to post-NF ROI activation of the *turbulence* > *non-turbulence* contrast by responder status. Gray lines connect paired data; filled violins = post-NF, empty = pre-NF. No significant changes in activation were found in either group. D) Violin plots showing changes in functional connectivity (PPI estimates on the *turbulence* > *non-turbulence* contrast) from the rTPJ seed to other ROIs from pre- to post-NF. A significant decrease in rTPJ-SMA connectivity was observed in non-responders (*p*_FDR-corr_ = 0.008), while responders showed a trend towards increased rTPJ-dlPFC connectivity (*p*_FDR-cor__r_ = 0.098). dlPFC, dorsolateral prefrontal cortex; IC, insular cortex; JoA, judgement of agency; lTPJ, left temporoparietal junction; NF, neurofeedback; PPI, psychophysiological interactions; Non-Resp., non-responder; Resp., responder; ROI, region of interest; rTPJ, right temporoparietal junction; SMA, supplementary motor area. ° *p* ≤ 0.1 (trend-level), * *p* < 0.05, ** *p* < 0.01, *** *p* < 0.001.

For the ROI analysis, mean contrast estimates (*turbulence* vs. *non-turbulence*) were extracted from predefined regions within the SoA network for each participant and session, including the rTPJ, lTPJ, SMA, IC, and dlPFC. The rTPJ ROI was identical to the mask used during the NF training, while the ROI for the lTPJ was created by mirroring the rTPJ mask to the left hemisphere (see Fig. 2B). The remaining ROIs − bilateral SMA, IC, and dlPFC − were defined according to the Glasser atlas (Glasser et al., 2016). Pre-to-post NF changes in ROI activity were assessed using two-sided paired *t*-tests for normally distributed data or Wilcoxon signed-rank tests for non-normally distributed data. Results were corrected for multiple comparisons using FDR correction at *q*_FDR_ = 0.05.

##### 2.5.3.2 Psychophysiological interaction analysis of the agency task

To assess changes in task-dependent functional connectivity, a psychophysiological interaction analysis (PPI) was performed. The rTPJ was used as the seed region, defined by the same mask as the one used for the NF training. The physiological regressor was the average BOLD time series extracted from the rTPJ, while the psychological regressor represented the task time course for the *turbulence* > *non-turbulence* contrast. The interaction term was computed as the element-wise product of the deconvolved seed signal and the task time course, reflecting condition-dependent modulation of connectivity with the rTPJ. First-level PPI models included physiological, psychological, and interaction terms, as well as movement outliers (FD > 0.5 mm). At the second level, changes in task-modulated rTPJ connectivity from visit 2 to visit 4 were analyzed using GLMs that included covariates of age, sex, and a composite mood (*z*-scored BDI and STAI-T). A validity check was performed by evaluating the overall PPI contrast across both sessions to confirm expected network engagement.

For the ROI-level PPI analysis, connectivity changes between the rTPJ and other predefined regions of interest (the lTPJ, SMA, IC, and dlPFC) were examined with two-sided paired *t*-tests. Analyses were conducted both at the group level and stratified by responder status. All results were corrected for multiple comparisons using FDR at *q*_FDR_ = 0.05.

##### 2.5.3.3 Neurofeedback training

At the first level, separate GLMs were specified for each of the nine NF runs. Each model included regressors for the regulation phases, modeled as blocks convolved with the canonical hemodynamic response function, and regressors for motion outliers (FD > 0.5 mm). At the second level, two complementary approaches were employed to evaluate brain activity during NF training. Firstly, a subject-level fixed-effects model was used to average activation across all runs. These individual activation maps were then entered into a second-level random-effects GLM to assess overall group-level effects, which served as a validity check of the brain activations of the NF training. Secondly, a full factorial model was used to evaluate changes in brain activation over time across the nine runs, capturing potential learning-related dynamics. For ROI analyses, mean beta estimates were extracted for each run and participant from each of the predefined ROIs within the SoA network (rTPJ, lTPJ, SMA, IC, and dlPFC). Linear mixed-effects models (LMMs) were fitted for each ROI, with participants modeled as random effects and run number as a fixed effect. Covariates included age, sex, and a composite mood score (*z*-scored BDI and STAI-T). In an additional model, binary psychotropic medication intake status was also added as a covariate. Finally, both whole-brain and ROI analyses were repeated separately for responders and non-responders to examine group-specific temporal dynamics and compare patterns of NF-related activation between the two subgroups.

#### 2.5.4 Association between neuroimaging measures, clinical, and behavioral scores

To examine associations between neuroimaging measures and changes in clinical or behavioral outcomes, a series of Spearman rank correlation analyses was performed. Neuroimaging measures included changes in ROI activation during the agency task, task-dependent functional connectivity (PPI), and changes in activation during NF sessions (comparing the final NF session to the first session). Clinical variables included changes in clinician-rated symptom severity (CGI, S-FMDRS), subjective symptom severity (SSS), depression (BDI), anxiety (STAI), as well as the three Neuro-QoL subscales (social role ability, social role satisfaction, cognitive function) and the physical and mental component scores of the SF-36. For each correlation, the corresponding *p*-value was computed and corrected for multiple comparisons using *q*_FDR_ = 0.05. Correlation coefficients are reported along with 95 % bias-corrected and accelerated (BCa) confidence intervals, calculated using 10,000 bootstrap resamples to ensure robust estimation.

## 3 Results

### 3.1 Clinical and demographic characteristics

Three patients were excluded post-hoc due to excessive motion artifacts during the NF training (defined as more than 30 % of frames with FD >0.5 mm). The final sample consisted of 18 FND patients (13 female, 5 male, mean age 35.4 ± 12.1 years). Baseline clinical and demographic characteristics at the first visit are summarized in Table 1. Predominant symptom types included motor weakness, sensorimotor impairment, gait disorder, and/or myoclonus, with a mean illness duration of 5.3 ± 4.2 years. Eleven patients (61 %) presented with a mixed symptom phenotype. At baseline, the sample reported mildly elevated depressive symptoms (BDI, 16 ± 10.2), moderately high trait anxiety (STAI-trait, 45.4 ± 13.6), and moderate levels of subjective symptom severity (49.2 ± 24.7), which is comparable with previously studied mixed FND cohorts (Sojka et al., 2021, Weber et al., 2025).

**Table 1:** Demographic and clinical characteristics of participants at baseline (visit 1, clinical pre-assessment). Continuous variables are presented as mean (standard deviation). Participants could present with more than one symptom type. BDI, Beck Depression Inventory; CGI, Clinical Global Impression Score; FND, functional neurological disorder; ICD-10, International Classification of Diseases 10th Edition; STAI, State-Trait Anxiety Inventory; S-FMDRS, Simplified Functional Movement Disorder Rating Scale.

Characteristic	FND (n = 18)
Age, years	35.4 (12.1)
Sex, female/male	13/5
Handedness, right/left/ambidexter	16/1/1
Symptom type	11 weakness 8 sensorimotor 7 gait disorder 5 myoclonus 4 seizures 2 tremor 1 dystonia 1 speech disorder
ICD-10 classification	11 mixed FND (F44.7), 5 motor FND (F44.4), 2 functional seizures(F44.5)
Duration of illness, years	5.3 (4.2)
Disease severity, CGI	3.1 (1.1)
Disease severity, S-FMDRS	8 (6.2)
Subjective symptom severity, SSS	49.2 (24.7)
Psychotropic medication	7 none 1 benzodiazepine 8 antidepressants 4 neuroleptics 2 antiepileptics 2 opioids
BDI	16 (10.2)
STAI	
Y1 (state)	38.1 (10.7)
Y2 (trait)	45.4 (13.6)

### 3.2 Neurofeedback feasibility

Overall, the NF training protocol was well tolerated. All 21 enrolled patients completed the full five-visit protocol with no dropouts. On a 5-point Likert scale, participants found the task moderately challenging (2.37 ± 1.0) and only mildly boring (1.7 ± 1.1). No serious adverse events occurred, and minor effects (e.g., headache, fatigue, dizziness) were mild, transient, and comparable to those typically reported in other MRI studies (Hawkinson et al., 2012). The most frequently reported effects are listed in Table S1. Subjective symptom severity (SSS), assessed immediately before and after each NF session, remained stable in sessions 1 and 3. A small but statistically significant increase was observed after session 2 (mean difference = 9.27 ± 13.3, *p* = 0.015; Cohen’s *d* = 0.32). However, this increase was not consistent across participants and was primarily driven by those later classified as responders. Importantly, symptom levels returned to baseline by the following session, suggesting that any increase in symptom severity was transient.

### 3.3 Behavioral outcomes

To assess the behavioral effects of NF training, we analyzed changes in the subjective ratings of agency (JoA), in which participants rated their perceived control over cursor movement. At the group level, participants reliably differentiated between *turbulence* and *non-turbulence* conditions both before and after training (Fig. 2A). Importantly, the magnitude of this difference increased significantly after training, suggesting an improved explicit SoA, i.e., a greater ability to distinguish between states of control and disrupted control.

However, individual responses varied considerably. While some participants showed substantial improvements in JoA differentiation, others exhibited minimal or no change at all. To capture this variability, effect sizes (Cohen’s *d*) were calculated for each participant’s change in JoA difference from pre- to post-training. Participants with large effect sizes (*d* ≥ 0.8) were classified as responders (n = 8), while those below this threshold were classified as non-responders (n = 10; see Supplementary Materials F for more details). For the secondary behavioral outcome, JoP, no significant pre-post changes were observed, though ratings remained significantly different between conditions (Fig. S2).

### 3.4 Neuroimaging outcomes

#### 3.4.1 Agency task

To evaluate whether the agency task successfully engaged the rTPJ and other regions of the agency network, we first pooled data across both the pre- and post-NF agency task. Whole-brain analysis of the *turbulence* > *non-turbulence* contrast revealed robust activation in key agency-related regions, including the rTPJ (T = 7.51), the right SMA (T = 6.79), and the precentral cortex (T = 6.75) (Supplementary Materials, Fig. S3 and Table S3), confirming the task’s construct validity.

ROI analyses of predefined agency-related regions (rTPJ, lTPJ, IC, SMA, dlPFC) revealed no significant change in activation from pre- to post-training, neither at the group level nor when stratified by responder status (Fig. 2B–C). Notably, rTPJ activity did not significantly increase in either subgroup following training.

In terms of task-based functional connectivity, pooled data across both sessions confirmed that the rTPJ was functionally connected with expected regions, including the SMA, prefrontal cortex, and cerebellum (Fig. S4; Table S4). ROI-based PPI analyses revealed a significant decrease in rTPJ–SMA connectivity among non-responders after training (*p*_FDR-corr_ = 0.008), whereas responders showed a trend-level decrease in rTPJ–dlPFC connectivity (*p*_FDR-corr_ = 0.098) (Fig. 2D).

The results of the resting-state functional connectivity analysis, which revealed no significant temporal changes, are presented in Supplementary Materials J.

#### 3.4.2 Neurofeedback training

**Fig. 3:**
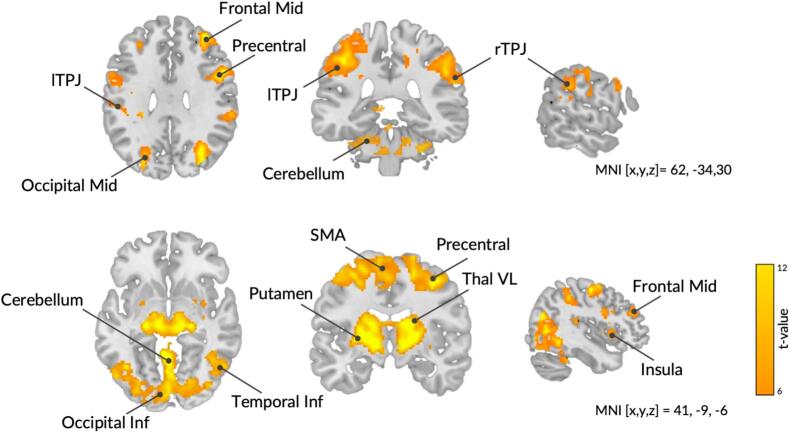
Brain activation patterns during neurofeedback training. Group-level whole-brain activation map for the contrast *upregulation > baseline* across all nine neurofeedback runs. For visualization, maps are thresholded at voxel-wise *p* < 0.001 (uncorrected) with cluster-level FWE correction (FWE_c_ = 37). At a voxel-wise FWE-corrected threshold, smaller clusters within these regions survived. Activation was observed in key areas implicated in agency and neurofeedback, including the lTPJ, rTPJ, SMA, cerebellum, anterior insula, putamen, and prefrontal cortex, supporting the construct validity of the paradigm. No significant deactivations were found for the reverse contrast. FWE, family-wise error; lTPJ, left temporoparietal junction; rTPJ, right temporoparietal junction; SMA, supplementary motor area.

To assess how the NF training task engaged the target network, we first examined group-level activation across all nine runs. Whole-brain analysis revealed robust activation in regions associated with the SoA (e.g., inferior parietal lobe overlapping the TPJ, SMA, and cerebellum), as well as areas commonly implicated in NF engagement and cognitive control (e.g., left anterior insula, putamen, and prefrontal cortex) (Fig. 3; Table S6), supporting the construct validity of the NF paradigm.

Next, a full factorial model was used to assess learning-related dynamics across the nine NF runs. No significant group-level changes in whole-brain activation were found, suggesting an absence of consistent linear training effects across participants.

**Fig. 4:**
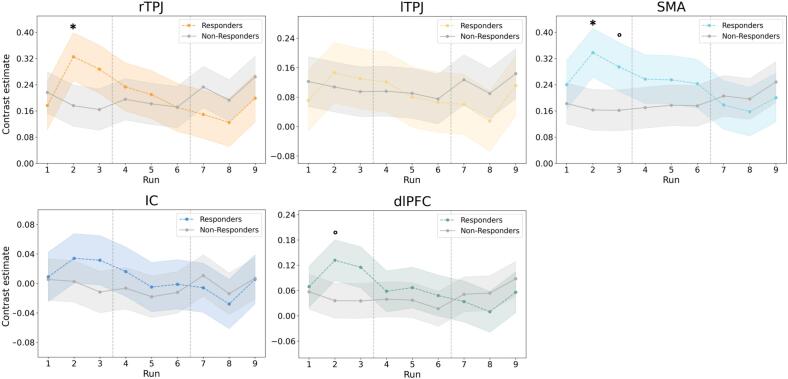
Training-related changes in ROI activity during neurofeedback. Contrast estimates (*upregulation > baseline*) across nine neurofeedback runs are shown separately for responders (n = 8) and non-responders (n = 10) in each of the ROI within the sense of agency network. Group means and 95 % confidence intervals are derived from marginal means of a linear mixed-effects model controlling for age, sex, depression, and anxiety. A mixed ANOVA revealed significant group × run interactions (see Table 2). Post-hoc pairwise comparisons showed significant group differences in run 2 for the rTPJ and SMA, with trend-level differences in run 2 for the dlPFC and run 3 for the SMA. dlPFC, dorsolateral prefrontal cortex; IC, insular cortex; lTPJ, left temporoparietal junction; ROI, region of interest; rTPJ, right temporoparietal junction; SMA, supplementary motor area. ° *p* < 0.1 (trend-level), * *p* < 0.05.

To investigate regional changes in activation, LMMs were fitted for each ROI. A mixed ANOVA on the fitted values revealed significant group-by-run interactions in the rTPJ, SMA, and dlPFC (Table 2), indicating that responders and non-responders exhibited different patterns of activation over time. Post-hoc comparisons revealed significant between-group differences in run 2 for the rTPJ (*p*_FDR-corr_ = 0.042) and SMA (*p*_FDR-corr_ = 0.015), with trend-level effects for the dlPFC (*p*_FDR-corr_ = 0.067, run 2) and SMA (*p*_FDR-corr_ = 0.06, run 3) (Fig. 4).

**Table 2:** Mixed ANOVA results for neural activity in the sense of agency network ROIs across neurofeedback runs. Results of a mixed ANOVA testing the effects of training run (1–9), group (responders vs. non-responders), and their interaction on neural activity within ROIs of the agency network. Analyses were adjusted for covariates (sex, age, depression, and anxiety scores). Effect sizes are reported as partial eta squared (**η_p_^2^**), where significant effects (*p* < 0.05, uncorrected) are shown in bold.

ROI	Term	F^a^	η_p_^2^
rTPJ	Run Group **Interaction**	1.95 0.21 3.95***	0.109 0.016 **0.198**
lTPJ	**Run** Group Interaction	2.4* 0.16 1.78	**0.130** 0.012 0.100
SMA	Run Group **Interaction**	1.49 2.44 5.15***	0.085 0.158 **0.243**
IC	**Run** Group Interaction	2.21* 0.48 1.51	**0.121** 0.036 0.081
dlPFC	**Run** Group **Interaction**	2.05* 1.01 3.23**	**0.114** 0.072 **0.168**

dlPFC, dorsolateral prefrontal cortex; IC, insular cortex; lTPJ, left temporoparietal junction; ROI, region of interest; rTPJ, right temporoparietal junction; SMA, supplementary motor area.

* *p* < 0.05, ** *p* < 0.01, *** *p* < 0.001.

a Degrees of freedom: Run = 2,128; Group = 1,13; Run × Group = 2,128.

Additionally, main effects of run were observed in the lTPJ, IC, and dlPFC, reflecting consistent changes in activity across time irrespective of responder status. When psychotropic medication status was included as an additional covariate alongside age, sex, and composite mood (*z*-scored BDI and STAI-T), the rTPJ difference in run 2 between responders and non-responders was attenuated to a trend level (*p*_FDR-corr_ = 0.069), whereas the SMA difference remained statistically significant (*p*_FDR-corr_ = 0.008).

### 3.5 Neurofeedback effect on clinical variables

Analysis of clinical outcome variables, including CGI and S-FMDRS scores, SSS, and questionnaire, revealed no statistically significant changes from pre- to post-NF training at the group level (Table 3). When examined separately, neither responders nor non-responders showed statistically significant clinical changes over time. At a descriptive level, responders showed a non-significant increase in subjective symptom severity following NF training (pre: 40.5 ± 26.7 vs. post: 49.25 ± 31.9, *p* = 0.11), while non-responders showed a slight decrease (pre: 56.1 ± 22.3 vs. post: 48.9 ± 30.4, *p* = 0.24).

**Table 3:** Comparison of clinical measures before and after neurofeedback training. Descriptive statistics are presented as mean (standard deviation). No comparison reached statistical significance. Effect sizes (Cohen’s *d*) reflect pre-post differences, with negative values indicating higher baseline scores. BDI, Beck Depression Inventory; CGI, Clinical Global Impression; Neuro-QoL, Quality of Life in Neurological Disorders; S-FMDRS, Simplified Functional Movement Disorder Rating Scale; SF-36, 36-Item Short Form Health Survey; SSS, Subjective Symptom Severity; STAI, State-Trait Anxiety Inventory.

Measure	Pre (M ± SD)	Post(M ± SD)	*t*-value	Effect size (*d*)
S-FMDRS	8 (6.2)	7.6 (5.8)	0.69	0.06
CGI	3.1 (1.1)	3.1 (1.1)	0	0
SSS	49.2 (24.9)	48.6 (31.5)	0.14	0.02
BDI	16 (10.2)	15.8 (10.6)	0.09	0.01
STAI				
Y1 (state)	38.1 (10.7)	36.4 (12.8)	0.96	0.15
Y2 (trait)	45.4 (13.6)	44.4 (13.2)	1.23	0.07
Neuro-QoL				
Ability social roles	23.8 (6.8)	23.9 (7.1)	−0.36	−0.01
Satisfaction social roles	23.1 (7.4)	23.6 (9.6)	−0.54	−0.07
Cognitive function	22.5 (7.7)	23.7 (9.6)	−0.08	−0.09
SF-36				
Physical component score (PCS)	38.7 (12.2)	38.4 (12.1)	0.16	0.02
Mental component score (MCS)	40.1 (10.4)	38.4 (10.8)	1.25	0.23

Baseline comparisons (i.e., at the first visit) between responders and non-responders revealed no statistically significant group differences on any clinical variable (Table S7). However, some numerical trends were observed. Responders tended to exhibit numerically higher objective motor symptom severity at baseline compared to non-responders, with higher average S-FMDRS scores (9.8 ± 7.6 vs. 6.6 ± 4.6, *p* = 0.3) and slightly higher CGI scores (3.4 ± 1.2 vs. 2.9 ± 1.1, *p* = 0.4). Conversely, responders reported lower subjective symptom burden than non-responders (40.5 ± 26.7 vs. 56.1 ± 22.3; *p* = 0.3).

### 3.6 Modulators of neurofeedback efficacy

#### 3.6.1 Cognitive strategies

To explore the role of individual cognitive strategies in neurofeedback response, we descriptively analyzed participant-reported strategies across training sessions. While responders tended to maintain task-focused approaches, non-responders increasingly shifted toward non-task-related strategies; however, no significant group differences were observed. Further details are provided in the Supplementary Materials M.

#### 3.6.2 Task performance

Task performance was assessed during both the NF training sessions and the agency task, defined as the difference between the percentage of targets hit and the percentage of distractors hit.

In the agency task, responders exhibited significant performance improvements over time in both the turbulence and non-turbulence conditions (Fig. S8), suggesting enhanced task engagement or learning. This improvement was not observed in non-responders. Notably, baseline performance in the first session did not differ significantly between the two groups, indicating that these differences likely emerged during or as a result of the training.

During the NF training paradigm, both responders and non-responders showed changes in performance across runs (Fig. S9). Linear mixed-effects modeling, controlling for age, sex, and baseline mood symptoms (BDI/STAI scores), revealed a significant main effect of run (*F*(8,180) = 3.55, *p* = 0.0009), indicating overall improvement across sessions. There was a trend-level main effect of group (*F*(1,19) = 4.48, *p* = 0.054), but the run-by-group interaction was not significant (*F*(8,180) = 1.63, *p* = 0.12). These findings suggest that while both groups showed some improvement over time, responders demonstrated more consistent gains across both the agency and NF tasks, potentially reflecting greater engagement with, or sensitivity to, the NF.

### 3.7 Neuroimaging-clinical-behavioral associations

For neuroimaging-behavioral associations, improvements in the SoA (indexed by increased JoA condition differentiation post–pre) were positively correlated with increased rTPJ–SMA task-based functional connectivity (Table S8; ρ = 0.55, uncorrected results). In contrast, JoA improvements were negatively correlated with changes in activation of the rTPJ (ρ = −0.55), SMA (ρ = −0.51), and IC (ρ = −0.54) across sessions (last vs. first NF session), suggesting that greater behavioral gains were associated with smaller increases (or decreases) in activation over time. Although these correlations did not survive correction for multiple comparisons, they are consistent with earlier group-level neuroimaging differences: responders showed early increases in rTPJ and SMA activation during the initial NF session, while non-responders exhibited reduced rTPJ–SMA connectivity post-training.

**Fig. 5:**
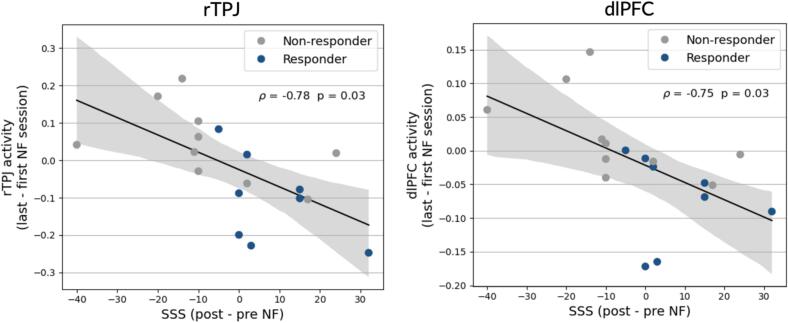
Correlations between clinical changes and neurofeedback-related activity changes in the full sample (n = 18). Significant Spearman correlations were observed between changes in ROI activation in the rTPJ (left) and dlPFC (right) during neurofeedback training (last session minus first session) and changes in subjective symptom severity (SSS; post minus pre-training). Responders are shown in blue, and non-responders are shown in gray. Shaded regions indicate 95 % confidence intervals. dlPFC, dorsolateral prefrontal cortex; ROI, region of interest; rTPJ, right temporoparietal junction. (For interpretation of the references to colour in this figure legend, the reader is referred to the web version of this article.)

No significant associations were found between changes in ROI activation during the agency task and clinical measures (Table S9). However, uncorrected associations in task-based connectivity revealed two trends: greater improvements in Neuro-QoL satisfaction were linked to reduced rTPJ–SMA connectivity (ρ = –0.57, *p* = 0.025), while declines in SF-36 mental component summary scores were associated with increased rTPJ–IC connectivity (ρ = 0.62, *p* = 0.01). These correlations did not survive FDR correction (Table S10). In contrast, neural changes during NF training were more robustly associated with clinical changes (Fig. 5, Table S11). After FDR correction, two correlations remained significant: increases in subjective symptom severity after NF training were linked to greater rTPJ activation (ρ = –0.78, *p*_FDR-corr_ = 0.03) and greater dlPFC activation (ρ = –0.75, *p*_FDR-corr_ = 0.03) during the first NF training session compared with the last.

## 4 Discussion

This proof-of-concept study evaluated the feasibility and preliminary efficacy of fMRI NF to modulate the SoA network in patients with FND. The protocol demonstrated high feasibility, as all participants completed the five-visit study without dropouts, and only mild, transient side effects were reported. Following NF training, participants showed a significant group-level increase in subjective agency ratings (JoA), consistent with the primary hypothesis. This improvement was primarily driven by a subset of participants classified as responders, who were distinguished by early neural activation patterns, with significantly greater activity in the rTPJ and SMA during the second run of the first NF session compared to non-responders. Despite these initial differences, neither responders nor non-responders showed consistent increases in activation across training runs or in the post-training agency task, suggesting limited consolidation or transfer of NF modulation. Task-based functional connectivity analyses revealed group-specific effects: non-responders displayed significant post-training decreases in rTPJ–SMA connectivity, whereas responders showed a trend-level decrease in rTPJ–dlPFC connectivity. Clinically, neither clinician-rated symptom severity nor subjective symptom burden showed significant improvement at the group level after training. However, exploratory correlation analyses revealed that reductions in self-reported symptom severity during training were significantly correlated with reduced activation of the rTPJ and dlPFC during the first NF session compared to the third.

### 4.1 Behavioral outcomes

At the behavioral level, we observed a significant group-level increase in JoA following NF training, indicating the overall effectiveness of our paradigm. This improvement was driven by a subset of participants (responders, n = 8) who showed large effect sizes, highlighting considerable inter-individual variability in behavioral responses. Increases in JoA following NF training were associated with higher rTPJ and SMA activation during the first NF training session compared to the last session. While these associations did not survive correction for multiple comparisons, they suggest that early modulation of key agency-related regions may play a role in supporting subsequent behavioral gains.

This observed increase in explicit agency contrasts with previous neuromodulation studies using the same agency task, which were able to modulate neural activity but failed to produce measurable behavioral changes. For example, a recent rTMS study targeting the rTPJ in both FND patients and healthy controls found no stimulation-specific effects on JoA, with both groups showing only non-specific improvements across sessions, likely due to task familiarity or repeated testing effects (Bühler et al., 2024). Similarly, a single-session EEG-based NF study in healthy individuals targeting the agency network reported no significant changes in JoA, although task performance improved, again suggesting practice effects rather than enhanced agency perception (Zito et al., 2023). Neither of these prior studies accounted for inter-individual variability or identified responders and non-responders, despite well-documented heterogeneity in both NF and broader neuromodulation outcomes (Alkoby et al., 2018, Haugg et al., 2021, Weber et al., 2011). It is therefore possible that meaningful JoA improvements may have occurred in a subset of participants but were obscured by averaging across the whole sample. Our findings highlight the importance of identifying individual response profiles, suggesting that behavioral gains in the SoA may be confined to certain specific individuals with FND.

### 4.2 Neural mechanisms and learning dynamics in fMRI neurofeedback

Although participants demonstrated group-level improvements in explicit agency following NF training, these behavioral gains were not accompanied by corresponding changes in brain activation during the post-training agency task, which functioned as a transfer condition without real-time feedback. Specifically, no significant pre-to-post differences were observed in activation within core agency-related regions (rTPJ, lTPJ, IC, SMA, dlPFC), either across the entire group or when comparing responders to non-responders.

Given the limited transfer of NF effects to the post-training agency task, it becomes essential to examine the learning dynamics during the training phase itself. Only responders, i.e., those who increased their explicit agency after NF, showed increases in rTPJ and SMA activity, along with a trend-level increase in dlPFC during the second run of the first NF session. This transient pattern of neural engagement can be interpreted in light of the dual-process theory of NF learning, which posits that effective self-regulation initially relies on top-down cognitive strategies facilitated by explicit feedback, and may subsequently transition into bottom-up reinforcement processes driven by implicit cues (Arns et al., 2017, Sitaram et al., 2017).

In our paradigm, participants received both explicit (intermittent visual cues) and implicit (task-based, not disclosed to participants) feedback. The early increases in rTPJ and SMA activation in responders likely reflect initial top-down engagement (Strehl, 2014). However, mismatches between the perceived in-game performance (i.e., successfully catching targets) and feedback scores may have caused confusion. For instance, when task performance was high but inconsistently mirrored in the feedback score (or vice versa), this discrepancy could have disrupted reinforcement, weakening the perceived link between strategy and outcome and potentially undermining participants’ motivation to learn (Evans et al., 2015). As top-down strategies became less effective, attention may have shifted to implicit cues. Yet, the implicit cues may have lacked sufficient clarity or salience to support bottom-up, model-free learning processes, therefore limiting sustained activation in the ROIs of the agency network.

Functional connectivity analyses further supported evidence for diverging learning trajectories. In responders, a trend-level reduction in rTPJ–dlPFC connectivity was observed post-training. Although similar connectivity decreases have previously been associated with impaired agency in FND patients (Baek et al., 2017, Wegrzyk et al., 2017), we interpret this reduction in responders as potentially adaptive, reflecting a transition toward more automatic, efficient processing of agency following successful engagement with NF training. This interpretation aligns with findings from other NF studies, where reduced prefrontal coupling following training was linked to consolidation and more effortless regulation (Ros et al., 2017, Yamashita et al., 2017). Additionally, our descriptive results support this finding, showing that responders consistently employed task-focused strategies throughout training, i.e., remained cognitively engaged with the task, and showed improved performance in the post-training agency task, unlike non-responders. This greater attentiveness and sustained engagement may have enabled responders to better utilize the provided feedback, facilitating more effective learning throughout the training sessions.

In contrast, non-responders showed a significant reduction in rTPJ–SMA connectivity, a pattern more consistent with maladaptive plasticity or disengagement (Thibault et al., 2018, Weber et al., 2011). This decoupling may reflect a breakdown in the coordination between motor intention and agency-related processing, particularly given previous findings of abnormal rTPJ–SMA connectivity in FND (Baek et al., 2017, Kühn et al., 2013). Qualitative reports support this interpretation: over time, non-responders gradually shifted away from task-related strategies in favor of more passive approaches (e.g., relaxation, mindfulness), especially in later sessions. Such shifts may indicate frustration or learned helplessness in response to a perceived lack of control over the feedback (Evans et al., 2015, Sorger et al., 2019). This disengagement may, in turn, have contributed to the weakening of the very circuits that the training aimed to target. Supporting this interpretation, greater reductions in rTPJ–SMA connectivity were associated with smaller post-training improvements in explicit agency, suggesting that this disconnection may impair integration of motor intentions with agency experience (Moore and Haggard, 2008).

Taken together, these findings emphasize the variability and complexity of NF learning. While early increases in rTPJ and SMA activity in responders suggest initial top-down engagement, lasting self-regulation may depend on bottom-up reinforcement and consistent, interpretable feedback. Rather than sustained increases in ROI activity, the effects of NF were reflected in subtle changes in functional connectivity, which supports the idea that the SoA is shaped by network dynamics rather than by activity in isolated regions. Responders maintained task engagement and showed trends towards more automatic processing, whereas non-responders displayed signs of disengagement and maladaptive neural changes, particularly a reduction in rTPJ–SMA connectivity. These divergent trajectories emphasize the importance of creating NF protocols tailored to the individual, with future studies focusing on identifying predictive markers of response and optimizing training conditions to support effective and lasting outcomes.

### 4.3 Clinical outcomes and neuroimaging-clinical associations

No significant clinical improvements were observed following NF training on either clinician-rated (CGI, S-FMDRS) or self-reported (SSS, questionnaires) measures, neither at the group level nor when considering responders and non-responders. These findings align with previous research suggesting that clinical improvements may lag behind neural self-regulation (Rance et al., 2018) and that sustained or repeated upregulation may be required for lasting therapeutic effects.

Responders tended to present with more severe motor symptoms at baseline but reported lower subjective distress than non-responders, potentially reflecting a dissociation between objective symptom severity and subjective symptom awareness (Adewusi et al., 2021). Following NF training, responders showed a descriptive increase in SSS scores, despite no corresponding change in clinician-rated motor symptoms. Interestingly, greater reductions in rTPJ and dlPFC activation across the NF training sessions (i.e., higher rTPJ and dlPFC activity in the first NF session compared to the last) were significantly associated with increased SSS scores post-training, reinforcing the qualitative trends observed among responders. These findings suggest that engaging in agency-focused NF and improving explicit agency may have paradoxically heightened participants’ awareness of their symptoms, leading to increased subjective reporting in responders without objective clinical deterioration.

Overall, these findings highlight the importance of considering symptom perception when evaluating SoA outcomes in FND. Enhanced explicit agency may serve as an important intermediate outcome, increasing self-awareness and potentially a critical first step toward meaningful clinical change.

### 4.4 Limitations and future directions

This study presents several limitations due to its nature as a proof-of-concept study. First, the absence of a sham or active control condition prevents clear attribution of observed effects to NF-specific mechanisms. Improvements may instead reflect nonspecific factors such as task repetition, engagement, expectancy, or placebo effects. The relatively small sample size, which was determined by the proof-of-concept nature of the study and limited resources rather than a formal power calculation, also reduces statistical power and generalizability, increasing the likelihood of both type I and type II errors. Subgroup analyses (responders vs. non-responders) provide interesting insights but should be interpreted cautiously given reduced power, potential overfitting, and the inclusion of some uncorrected trends. Second, aspects of the training protocol may have limited participants’ ability to sustain self-regulation. The one-week interval between sessions may not have supported consolidation (Strehl, 2014), while repeated sessions could have led to fatigue or reduced motivation in fast learners (Sulzer et al., 2013). Prior studies suggest that self-regulation can sometimes be achieved within a single session, with limited benefits from extended protocols (Bauer et al., 2020, De Filippi et al., 2022, MacDuffie et al., 2018, Zito et al., 2023). Moreover, performing the agency task while simultaneously attempting to identify effective self-regulation strategies may have imposed excessive cognitive load, impairing NF learning in some individuals (Bauer and Gharabaghi, 2015). Furthermore, the adaptive NF design, whereby the task switched to non-turbulence only when rTPJ activity exceeded a threshold, may have inadvertently contributed to a reduced SoA in some participants. Those unable to increase rTPJ activity mostly remained in turbulence mode throughout training, receiving minimal positive feedback and potentially becoming disengaged from the task. Third, several measurement-related factors constrain interpretation. Behavioral assessments of JoA were limited to only 11 trials per condition and session, which may have reduced the reliability of individual scores. Because responder classification was derived from these ratings, measurement noise may have led to misclassification and obscured group-level comparisons. Transfer effects were assessed only before and after training (first vs. last NF session), whereas intermediate assessments would have offered a more granular view of learning trajectories and consolidation. In addition, the setup of the NF training task differed slightly from the post-training agency task (longer game phase, adaptive conditions), potentially limiting the latter’s sensitivity as a transfer measure. Finally, clinical and anatomical considerations may have influenced outcomes. The FND sample was clinically heterogeneous in symptom type and severity, likely contributing to variability in neural and behavioral responses. Responder status could not be predicted from clinical phenotype or illness duration, reducing insights into who might benefit most from NF. Furthermore, the rTPJ ROI was defined using healthy control data (*turbulence* > *non-turbulence*) rather than FND-specific hypoactivation patterns, which are difficult to delineate with a functional localizer. While this ensured targeting of a canonical agency-related region, it may have reduced training specificity for this population.

Future studies should employ sham-controlled designs with larger, more homogeneous samples, ideally stratified by symptom profile (e.g., patients with predominant motor symptoms) or by baseline neural markers such as resting-state connectivity patterns or structural measures. Additionally, assessing a broader range of demographic and clinical characteristics (e.g., educational level, cognitive measures) could provide valuable insight into the traits distinguishing responders from non-responders. Tracking agency-related activation, task engagement, and self-reported strategy use after each session would help to better map learning trajectories and identify early predictors of treatment response. Here, given that consistent, task-focused strategies were linked to better outcomes, future protocols may benefit from providing structured, task-specific instructions. Incorporating more nuanced adaptive task difficulty adjustments (beyond the current two levels) and gamified elements could further boost engagement, motivation, and transfer of training effects. Finally, systematically probing the neural and behavioral mechanisms underlying individual variability will be key to refining protocols and developing more effective, personalized clinical NF interventions.

## 5 Conclusion

This proof-of-concept study demonstrated that fMRI NF of the rTPJ is feasible in FND and can enhance explicit agency. Behavioral gains were driven by a subset of responders who exhibited early, transient increases in rTPJ and SMA activation during training, as well as subtle post-training connectivity shifts suggestive of adaptive network reconfiguration. These neural changes occurred alongside increased self-reported symptom severity but without corresponding changes in clinician-rated measures, raising the possibility that enhanced agency may heighten symptom awareness rather than immediately reduce symptoms. In contrast, non-responders showed signs of disengagement and maladaptive connectivity changes, most notably reduced rTPJ–SMA coupling, without evidence of clinical deterioration. Descriptive observations suggested that higher baseline motor symptom severity, lower subjective distress, and sustained task engagement may be associated with greater NF responsiveness. Together, these findings suggest that NF can modulate agency-related circuits in a subset of FND patients, while highlighting the variability of individual responses and the need to identify robust predictors of benefit. Future sham-controlled trials with larger samples, optimized feedback and transfer assessments, and longitudinal engagement monitoring will be essential to determine who benefits and under what conditions, paving the way for personalized NF interventions in FND.

## Declaration of Generative AI and AI-assisted technologies in the writing process

During the preparation of this work, the authors used OpenAI’s ChatGPT (GPT-4 turbo) to enhance readability. After using this tool/service, the authors reviewed and edited the content as needed and take full responsibility for the content of the published article.

## CRediT authorship contribution statement

**Eliane Müller:** Data curation, Formal analysis, Investigation, Methodology, Project administration, Software, Validation, Visualization, Writing – original draft, Writing – review & editing. **Serafeim Loukas:** Conceptualization, Data curation, Investigation, Methodology, Software, Validation. **Salome Häuselmann:** Data curation, Investigation, Writing – review & editing. **Cristina Concetti:** Writing – review & editing. **Dimitri Van De Ville:** Conceptualization, Methodology, Resources, Supervision, Writing – review & editing. **Nicolas Gninenko:** Conceptualization, Data curation, Formal analysis, Investigation, Methodology, Software, Supervision, Validation, Writing – review & editing. **Selma Aybek:** Conceptualization, Funding acquisition, Investigation, Resources, Supervision, Writing – review & editing.

## Funding

This work was supported by the Fondation de Préfargier.

## Declaration of competing interest

The authors declare that they have no known competing financial interests or personal relationships that could have appeared to influence the work reported in this paper.

## Data Availability

Due to the terms of the participants’ informed consent, data sharing is not permitted. The code used for all data analyses can be found here: https://github.com/FND-ResearchGroup/fMRI_NF_FND.
