# Supplementary material for: Modulating the sense of agency in functional neurological disorder using real-time fMRI neurofeedback: a proof-of-concept study
Source: Neuroimage Clin. 2025 Nov 2;48:103899. doi: 10.1016/j.nicl.2025.103899 (PMC12637265; doi:10.1016/j.nicl.2025.103899)
Supplement: Supplementary Data 1 [file mmc1.docx]

Supplementary material to: Modulating the sense of agency in functional neurological disorder using real-time fMRI neurofeedback

Eliane Müller^a,b,c,d^, Serafeim Loukas^b,c,e^, Salome Häuselmann^a,c,d^, Cristina Concetti^a^, Dimitri Van De Ville^b,e^, Nicolas Gninenko^a,b,c,e,1^, Selma Aybek^a,1^

^a^ Department of Neurology, Faculty of Science and Medicine, University of Fribourg, Switzerland

^b^ Neuro-X Institute, Ecole Polytechnique Fédérale de Lausanne (EPFL), Geneva, Switzerland

^c^ Department of Neurology, Psychosomatic Medicine Unit, Inselspital Bern University Hospital, Switzerland

^d^ Graduate School of Cellular and Biomedical Sciences (GCB), University of Bern, Switzerland

^e^ Department of Radiology and Medical Informatics, University of Geneva, Switzerland

^1^ These authors share last authorship

**Corresponding authors:**

Dr. Nicolas Gninenko, Medical Image Processing Lab, Neuro-X Institute, Ecole Polytechnique Fédérale de Lausanne (EPFL), Chemin des Mines 9, 1202 Geneva, Switzerland; [nicolas.gninenko@gmail.com](mailto:nicolas.gninenko@gmail.com)

Prof. Dr. med. Selma Aybek, Department of Neurology, Faculty of Science and Medicine, University of Fribourg, Chemin du Musée 5, 1700 Fribourg, Switzerland; [selma.aybek@unifr.ch](mailto:selma.aybek@unifr.ch)

Consensus on the Reporting and Experimental Design of clinical and cognitive-behavioural Neurofeedback studies (CRED-nf) best practices checklist 2020* (Ros et al., 2020)

| Domain | Item # | Checklist item | Reported in Section(s) |
| --- | --- | --- | --- |
| Pre-experiment | | | |
|  | 1a | Pre-register experimental protocol and planned analyses | 2.1 |
|  | 1b | Justify sample size | 4.4 |
| Control groups | | | |
|  | 2a | Employ control group(s) or control condition(s) | 2.4.2 |
|  | 2b | When leveraging experimental designs where a double-blind is possible, use a double-blind | N/A |
|  | 2c | Blind those who rate the outcomes, and when possible, the statisticians involved | N/A |
|  | 2d | Examine to what extent participants and experimenters remain blinded | N/A |
|  | 2e | In clinical efficacy studies, employ a standard-of-care intervention group as a benchmark for improvement | N/A |
| Control measures | | | |
|  | 3a | Collect data on psychosocial factors | 2.2 |
|  | 3b | Report whether participants were provided with a strategy | 2.4.2 |
|  | 3c | Report the strategies participants used | 3.6.1 |
|  | 3d | Report methods used for online-data processing and artifact correction | 2.4.2 |
|  | 3e | Report condition and group effects for artifacts | N/A |
| Feedback specifications | | | |
|  | 4a | Report how the online-feature extraction was defined | 2.4.2 |
|  | 4b | Report and justify the reinforcement schedule | 2.4.2 |
|  | 4c | Report the feedback modality and content | 2.4.2 |
|  | 4d | Collect and report all brain activity variable(s) and/or contrasts used for feedback, as displayed to experimental participants | 2.4.2 |
|  | 4e | Report the hardware and software used | 2.4.2,2.5, Supplementary Materials A |
| Outcome measures | | | |
| Brain | 5a | Report neurofeedback regulation success based on the feedback signal | 3.4.2 |
|  | 5b | Plot within-session and between-session regulation blocks of feedback variable(s), as well as pre-to-post resting baselines or contrasts | 3.4 |
|  | 5c | Statistically compare the experimental condition/group to the control condition(s)/group(s) (not only each group to baseline measures) | N/A |
| Behaviour | 6a | Include measures of clinical or behavioural significance, defined a priori, and describe whether they were reached | 3.3, 3.5 |
|  | 6b | Run correlational analyses between regulation success and behavioural outcomes | 3.7 |
| Data storage | | |  |
|  | 7a | Upload all materials, analysis scripts, code, and raw data used for analyses, as well as final values, to an open access data repository, when feasible | Section on ‘Data availability’ |

*Darker shaded boxes represent *Essential* checklist items; lightly shaded boxes represent *Encouraged* checklist items.

## fMRI data acquisition

High-resolution anatomical images were collected using a T1-weighted magnetization prepared rapid gradient echo (MPRAGE) sequence (sagittal orientation, repetition time (TR) = 2300 ms, echo time (TE) = 2.25 ms, flip angle (FA) = 8°, voxel size = 1.0 mm^3^, FoV = 215 mm, matrix size = 208 x 256 x 256 (208 volumes, ~ 5 min). Functional data from the fMRI NF training task runs (380 volumes per run, 5 dummy scans in the beginning and at the end, 9.5 min), the agency task (~350 volumes, 8.75 min) and the resting-state fMRI sequence (320 volumes, 8 min) were acquired using an interleaved multi-slice echo-planar imaging (EPI) sequence (axial orientation, TR = 1500 ms, TE = 31 ms, FA = 64°, voxel size = 2.0 mm^3^, matrix size = 108 x 108 x 64). The agency tasks were implemented using MATLAB (R2022a, MathWorks Inc., USA) and Psychophysics Toolbox Version 3 (PTB-3) (Kleiner et al., 2007). Physiological data were also recorded during all fMRI acquisitions, with respiration monitored using a breathing belt and heart rate measured via photoplethysmography. Data were collected using a BIOPAC MP150 system (RSP100C amplifier; BIOPAC Systems, Inc., Goleta, USA) and AcqKnowledge software (version 4.4.1) for subsequent offline analysis. Participants were instructed not to move, except for their hand movements required for the task.

## Cognitive strategies underlying neurofeedback regulation

A descriptive content analysis of participants’ self-reported strategies, collected after each of the three NF training sessions, identified 12 distinct strategy categories (Figure S6). These categories were not predefined but derived directly from the content of the participants’ responses, based on thematic patterns. Strategies were then grouped into two broad types: task-related (e.g., focusing on the Xs, anticipating movement timing) and non-task-related (e.g., relaxation, mindfulness techniques). We documented the number of responders and non-responders who reported using each strategy category and calculated the total number of different strategies used by each participant across sessions.

## Offline preprocessing of fMRI data

Functional MRI data were preprocessed using SPM12 (Wellcome Centre for Human Neuroimaging, UCL, London). Structural T1 images were segmented into tissue types with simultaneous bias correction to remove intensity inhomogeneities caused by the scanner or participant anatomy. Functional images were slice-timing corrected, realigned to the mean image, and co-registered with the bias-corrected structural image. Physiological data (heart rate and respiration) were preprocessed with the PhysIO TAPAS toolbox (Kasper et al., 2017). Nuisance regressors included white matter and cerebrospinal fluid signals, six head motion parameters, physiological noise, and the global mean signal. Functional data were normalized to MNI space via linear registration to the anatomical image and spatially smoothed using a 6 mm full width at half-maximum (FWHM) Gaussian kernel. The Brain Extraction Tool (BET) (Smith, 2002) was applied to the smoothed, normalized mean functional volume to remove non-brain tissue and generate a binary mask.

1. Resting-state analysis

Resting-state seed-to-voxel functional connectivity analyses were conducted using the CONN toolbox (version 22a) (Whitfield-Gabrieli and Nieto-Castanon, 2012). Preprocessing was carried out in SPM and MATLAB as described previously, and the resulting data were imported into CONN for analysis. The same rTPJ seed mask used during fMRI neurofeedback training was applied to all participants. At the first level, seed-based connectivity (SBC) maps and ROI-to-ROI connectivity matrices were computed to quantify functional coupling between the rTPJ and the rest of the brain. Functional connectivity strength was expressed as Fisher-transformed bivariate correlation coefficients derived from a weighted general linear model (weighted GLM), modeling the association between the BOLD time series of the seed and target regions. Individual scans were weighted by a boxcar function representing each experimental condition, convolved with the canonical hemodynamic response function, and rectified. At the group level, voxel-wise random-effects GLMs were estimated with first-level connectivity maps as dependent variables and group or session identifiers as predictors. Cluster-level inferences were based on Gaussian Random Field theory, using a voxel-wise cluster-forming threshold of *p* < 0.001 (uncorrected) and a cluster-level familywise error correction threshold of *p*_FWE_ < 0.05.

## Summary of adverse events reported after the NF sessions

| Adverse event | Session 1 | Session 2 | Session 3 |
| --- | --- | --- | --- |
| Headache | 4 | 4 | 4 |
| Fatigue | 3 | 4 | 3 |
| Dizziness | 3 | 3 | 2 |
| Back / neck pain | 3 | 1 | 1 |
| Musculoskeletal stiffness | 1 | 2 | 2 |
| Anxiety | 2 | 1 | 0 |
| Tingling / buzzing sensation | 0 | 0 | 1 |

##

Table S1: Number of participants (out of 18) reporting each type of adverse event during each neurofeedback session. Most adverse events were mild and transient in nature. Two moderate events were reported: one case of dizziness in session 3 and one case of headache in session 2. All reported events resolved without medical intervention.

## Classification of responders and non-responders

To define responders and non-responders based on behavioral outcomes, we first examined the distribution of JoA difference scores (post–pre-NF) across participants (Fig. S1 A). This distribution was unimodal, though slightly skewed, indicating no clear natural split in the data. To identify participants with a clinically meaningful benefit, we set a threshold for the raw JoA difference at ≥ 1.7 points (post–pre). Additionally, we calculated individual effect sizes (Cohen’s *d*) for each participant’s change score (Lakens, 2013), applying a threshold of *d* = 0.8, consistent with a large effect (Fig. S1 B). Both criteria converged, identifying the same set of participants as responders, i.e., those who demonstrated both a raw score increase of ≥ 1.7 and an effect size of ≥ 0.8 (see Table S2). This dual-threshold approach ensured that improvements were both statistically robust and practically meaningful.


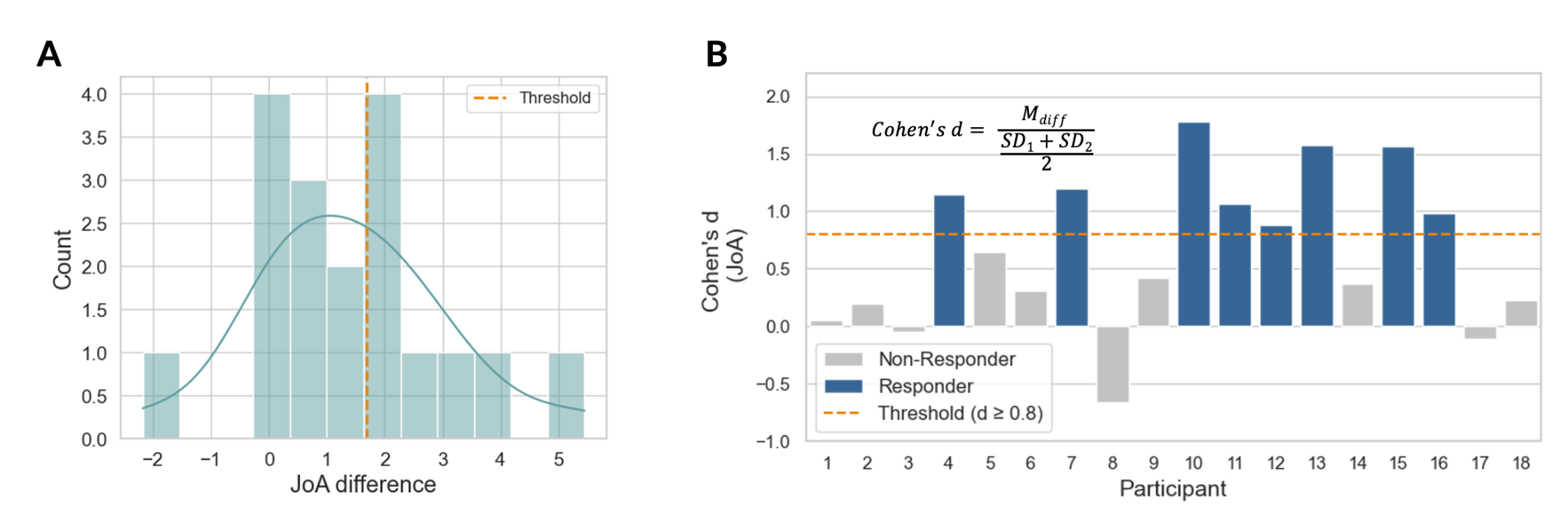


Fig. S1: Classification of responders and non-responders based on individual changes in Judgement of Agency (JoA). Participants were classified as responders (n = 8) if their JoA condition difference (turbulence vs. non-turbulence) increased by ≥ 1.7 points from pre- to post-NF training and the corresponding effect size (Cohen’s *d*) was ≥ 0.8, indicating a substantial improvement in agency judgments. All other participants (n = 10) were classified as non-responders.

| Participant # | JoA difference  (raw score) | JoA difference (Cohen’s *d*) | Responder (True/False) |
| --- | --- | --- | --- |
| 1 | 0.09 | 0.05 | False |
| 2 | 0.36 | 0.2 | False |
| 3 | -0.09 | -0.05 | False |
| 4 | 2.82 | 1.15 | True |
| 5 | 1.65 | 0.65 | False |
| 6 | 0.72 | 0.31 | False |
| 7 | 2.0 | 1.2 | True |
| 8 | -2.18 | -0.66 | False |
| 9 | 0.45 | 0.42 | False |
| 10 | 2.0 | 1.78 | True |
| 11 | 2.9 | 1.1 | True |
| 12 | 2,0 | 0.88 | True |
| 13 | 5.45 | 1.57 | True |
| 14 | 1.18 | 0.37 | False |
| 15 | 3.63 | 1.57 | True |
| 16 | 1.72 | 0.99 | True |
| 17 | -0.09 | -0.11 | False |
| 18 | 0.45 | 0.23 | False |

Table S2: Individual Judgement of Agency (JoA) differences (raw score and effect size) for each participant. Participants were classified as responders if their raw JoA difference (post- minus pre-NF) was ≥ 1.7 and the corresponding effect size (Cohen’s *d*) was ≥ 0.8, reflecting a large improvement in JoA scores.

## Judgement of Performance (JoP)


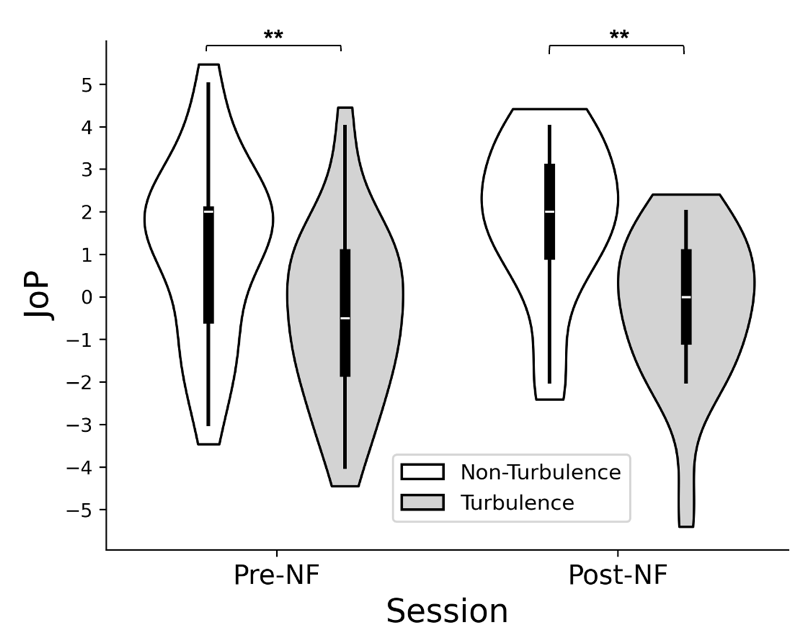


Fig. S2: Group-level judgment of performance (JoP) across all participants (n = 18), comparing subjective ratings between turbulence and non-turbulence conditions before and after neurofeedback (NF) training. A significant difference was observed between conditions at both time points (pre-NF: *p* = 0.0012; post-NF: *p* = 0.0013).

##
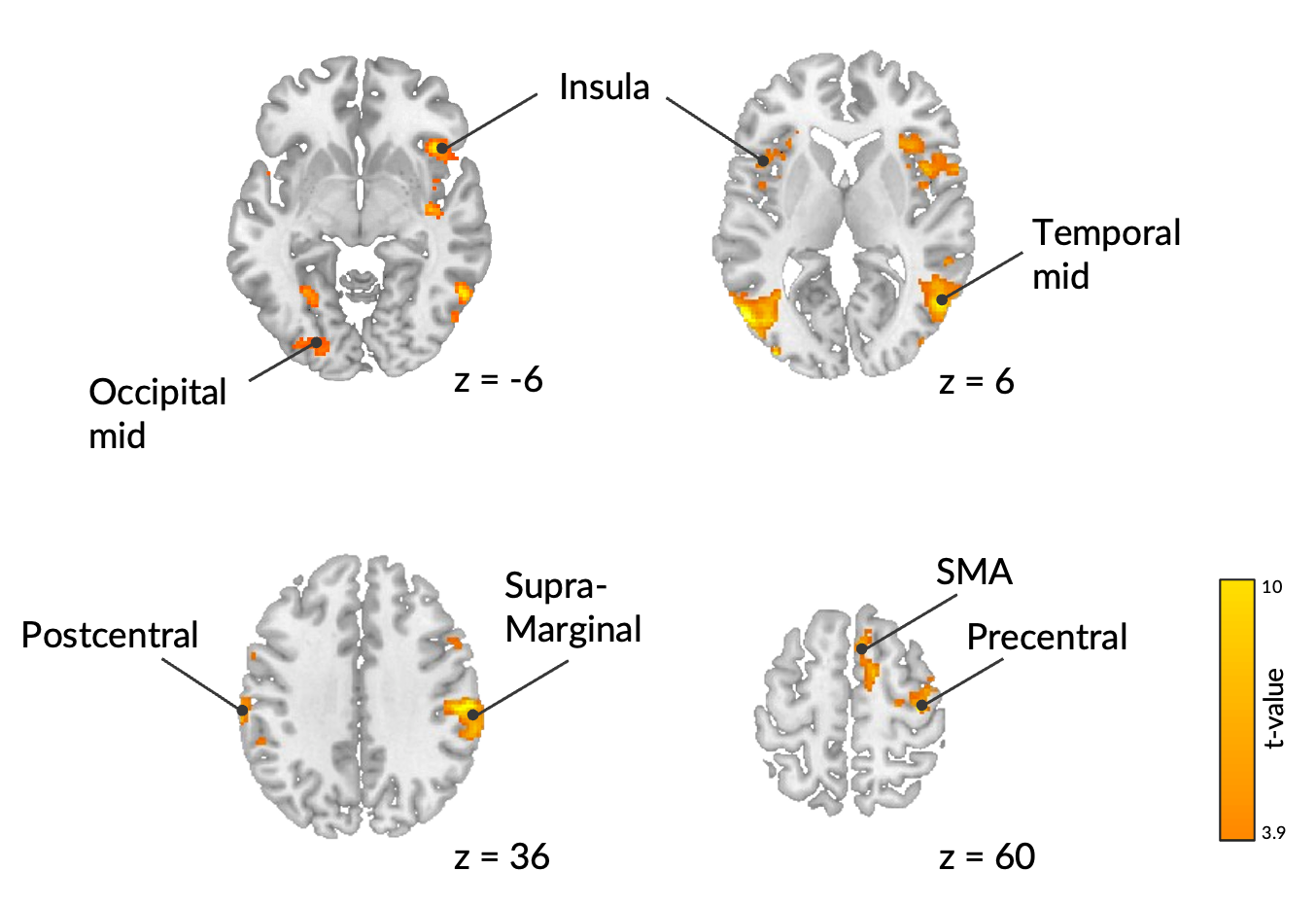
Whole-brain neuroimaging analysis of the agency task

Fig. S3: Neural correlates of the sense of agency during the agency task. Whole-brain analysis of the *turbulence > non-turbulence* contrast across both sessions (pre- and post-neurofeedback (NF)) revealed activations in key regions of the sense of agency network, including the bilateral insula, bilateral supramarginal gyrus, right precentral gyrus, left postcentral gyrus, and right supplementary motor area (SMA). The cluster in the right supramarginal gyrus overlapped with the rTPJ mask used for the NF training. Results are shown at an uncorrected voxel-wise threshold of *p* < 0.001 with cluster-level family-wise error (FWE) correction (FWE_c_ = 81 voxels). No significant differences were detected between responders and non-responders, and no clusters survived voxel-wise FWE correction. Regions are labelled according to the AAL3 atlas (Rolls et al., 2020).

|  | | peak-level | cluster-level | |
| --- | --- | --- | --- | --- |
| Region | MNI-Coordinates | T | p_FWE-corr_ | k_E_ |
| Frontal_Inf_Oper_R | [56 14 18] | 10.0 | <0.001 | 1177 |
| Insula_R | [40 20 -4] | 6.88 |  |  |
| Frontal_Inf_Oper_R | [52 6 26] | 6.43 |  |  |
| Occipital_Sup_R | [26 -94 14] | 9.47 | <0.001 | 222 |
| Occipital_Mid_R | [36 -88 16] | 7.15 |  |  |
| Occipital_Mid_R | [42 -82 0] | 4.48 |  |  |
| Postcentral_L | [-56 -22 26] | 8.67 | <0.001 | 619 |
| SupraMarginal_L | [-58 -32 26] | 6.71 |  |  |
| SupraMarginal_L | [-50 -32 28] | 5.87 |  |  |
| Temporal_Mid_L | [-52 -72 12] | 8.15 | <0.001 | 740 |
| Postcentral_R | [58 -20 34] | 7.99 | <0.001 | 2004 |
| Temporal_Mid_R | [48 -60 0] | 7.51 |  |  |
| Temporal_Inf_R | [56 -60 -4] | 7.22 |  |  |
| Supp_Motor_Area_R | [12 0 62] | 6.79 | <0.001 | 200 |
| Precentral_R | [36 -18 60] | 6.75 | <0.001 | 312 |
| Frontal_Mid_2_R | [38 2 42] | 5.65 |  |  |
| Precentral_R | [38 -10 62] | 5.21 |  |  |
| Vermis_6 | [0 -72 -12] | 6.66 | <0.001 | 598 |
| Cerebelum_4_5_L | [-22 -50 -20] | 6.61 | <0.001 | 416 |
| Insula_L | [-46 6 -2] | 6.56 | <0.001 | 452 |
| Rolandic_Oper_L | [-42 -6 14] | 6.38 |  |  |
| Insula_L | [-34 -4 12] | 6.13 |  |  |
| Occipital_Mid_L | [-38 -90 6] | 6.44 | <0.001 | 213 |
| Postcentral_L | [-56 -16 48] | 5.93 | <0.001 | 134 |
| Parietal_Inf_L | [-58 -30 50] | 4.52 |  |  |
| Postcentral_L | [-42 -22 44] | 4.38 |  |  |
| Insula_R | [38 -12 -6] | 5.73 | 0.014 | 104 |
| Fusiform_R | [30 -78 -8] | 5.6 | 0.023 | 93 |
| Precentral_L | [-26 -26 64] | 5.35 | 0.026 | 91 |
| Precentral_L | [-22 -18 64] | 5.16 |  |  |
| Postcentral_L | [-30 -32 68] | 4.36 |  |  |
| Postcentral_R | [26 -38 52] | 4.9 | 0.043 | 81 |
|  |  |  |  |  |

Table S3: Whole-brain analysis of the agency task activity across both sessions (pre- and post-neurofeedback) of the *turbulence* > *non-turbulence* contrast. The analysis included age, sex, depression, and anxiety scores as covariates. Results are reported at an uncorrected voxel-wise threshold of *p* < 0.001. While no clusters survived voxel-wise family-wise error (FWE) correction, the table lists clusters significant at the cluster-level FWE-corrected threshold (FWE_c_ = 81 voxels). Regions are labelled according to the AAL3 atlas (Rolls et al., 2020). k_E_, cluster extent.

## Whole-brain functional connectivity analysis of the agency task
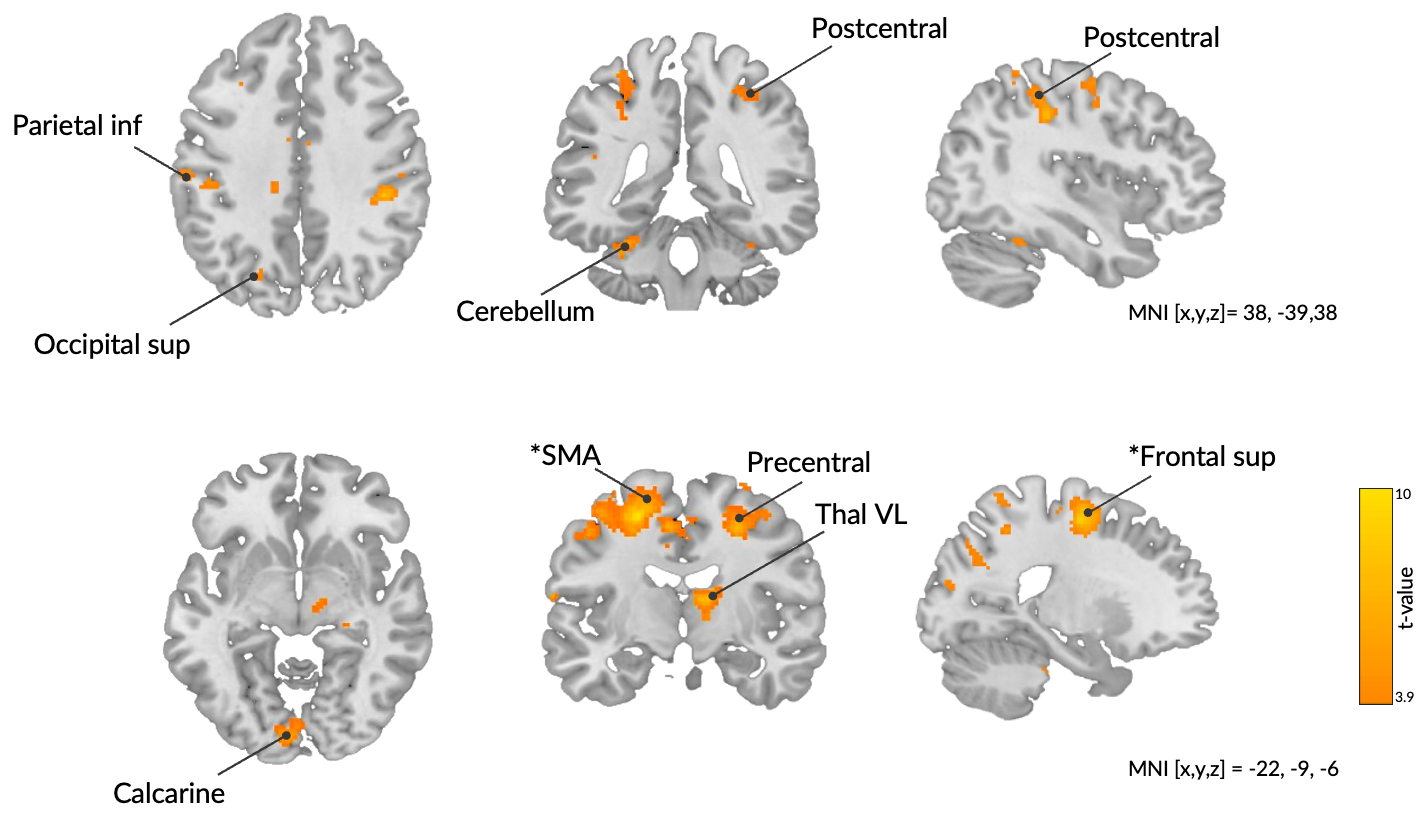


Fig. S4: Whole-brain functional connectivity analysis of the agency task. Task-related functional connectivity of the rTPJ (pre- and post-neurofeedback combined) revealed links with key regions associated with the sense of agency, including the supplementary motor area (SMA), postcentral cortex, and cerebellum. Results are displayed at an uncorrected voxel-wise threshold of *p* < 0.001 with cluster-level family-wise error (FWE) correction (FWE_c_ = 85 voxels). No subgroup differences emerged. At the voxel-wise FWE-corrected level (p < 0.05), only one cluster in the left superior frontal gyrus/SMA survived (marked with an asterisk (*)). Regions are labelled according to the AAL3 atlas (Rolls et al., 2020).

|  | | peak-level | cluster-level | |
| --- | --- | --- | --- | --- |
| Region | MNI-Coordinates | T | p_FWE-corr_ | k_E_ |
| Frontal_Sup_2_L | [-22 -10 56] | 6.83 | <0.001 | 3727 |
| Postcentral_L | [-46 -18 56] | 6.47 |  |  |
| Supp_Motor_Area_L | [-6 -12 50] | 6.21 |  |  |
| Cerebelum_4_5_L | [-30 -40 -26] | 5.69 | 0.01 | 122 |
| Cerebelum_4_5_L | [-24 -32 -28] | 4.89 |  |  |
| Cerebelum_6_L | [-30 -48 -24] | 3.68 |  |  |
| Postcentral_R | [32 -30 50] | 5.57 | <0.001 | 591 |
| Postcentral_R | [38 -30 40] | 5.20 |  |  |
| Postcentral_R | [52 -26 42] | 5.12 |  |  |
| Thal_VL_R | [12 -10 12] | 5.48 | <0.001 | 278 |
| Thal_MDl_R | [8 -18 4] | 5.31 |  |  |
| Thal_PuA_R | [12 -22 12] | 4.28 |  |  |
| Cerebelum_4_5_R | [14 -52 -16] | 5.30 | <0.001 | 393 |
| Cerebelum_4_5_R | [32 -32 -30] | 5.20 |  |  |
| Cerebelum_4_5_R | [28 -52 -22] | 5.07 |  |  |
| Calcarine_L | [-8 -88 -6] | 4.94 | 0.002 | 158 |
| Lingual_L | [-14 -80 0] | 3.83 |  |  |
| Calcarine_L | [-2 -82 -6] | 3.67 |  |  |
| Thal_VPL_L | [-12 -18 12] | 4.84 | 0.03 | 97 |
| Occipital_Sup_L | [-20 -68 36] | 4.67 | 0.002 | 168 |
| Parietal_Sup_L | [-18 -70 52] | 4.51 |  |  |
| Occipital_Sup_L | [-22 -62 28] | 3.95 |  |  |
| Occipital_Sup_L | [-26 -74 20] | 4.64 | 0.026 | 100 |
| Occipital_Mid_L | [-22 -84 18] | 4.42 |  |  |
| Parietal_Sup_R | [24 -56 48] | 4.22 | <0.001 | 242 |
| Parietal_Sup_R | [14 -64 58] | 4.21 |  |  |
| Occipital_Sup_R | [26 -66 42] | 4.20 |  |  |

Table S4: Whole-brain analysis of agency task-related functional connectivity across both sessions (pre- and post-neurofeedback). The model controlled for age, sex, depression, and anxiety scores. Results are reported at an uncorrected voxel-wise threshold of *p* < 0.001 and a cluster-level family-wise error (FWE)-corrected threshold (FWE_c_ = 85 voxels). Only the first cluster, located in the left superior frontal gyrus, survived voxel-wise FWE correction (*p* < 0.05). Regions are labelled according to the AAL3 atlas (Rolls et al., 2020). k_E_, cluster extent.

1. Resting-state functional connectivity results

As an exploratory analysis, resting-state functional connectivity was examined using the rTPJ as the seed. In the seed-to-voxel analysis, no significant session effects were detected at the group level, even at uncorrected thresholds. When data from all sessions were pooled, small uncorrected clusters in the right middle frontal gyrus and left middle temporal gyrus showed differences between responders and non-responders (see Fig. S5, Table S5). In the ROI-to-ROI analysis, no significant connectivity changes were observed over time or between groups, even at the uncorrected level.


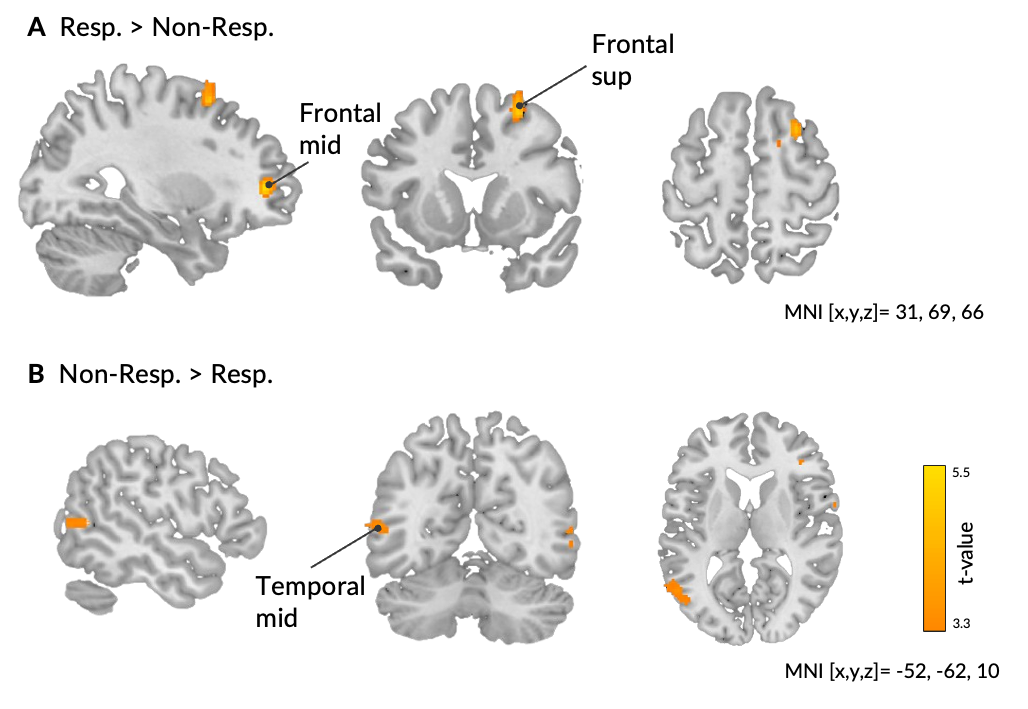


Fig. S5: Seed-to-voxel resting-state functional connectivity analysis using rTPJ as seed region. A) Responders showed greater rTPJ connectivity than non-responders in middle and superior frontal cortex clusters across all three sessions. B) Non-responders showed greater rTPJ connectivity than responders in the left middle temporal cortex across all sessions. Results thresholded at T = 3.3 (*p* < 0.001, uncorrected).

| Responders > Non-responders |  |  | peak-level | cluster-level | |
| --- | --- | --- | --- | --- | --- |
| Region | MNI-Coordinates | T | P _FWE-corr_ | k_E_ |  |
| Frontal_Mid_2_R | [32 52 4] | 5.98 | 0.067 | 349 |  |
| Frontal_Mid_2_R | [36 58 -4] | 5.56 | 0.07 |  |  |
| Frontal_Sup_2_R | [30 56 16] | 3.46 | 0.908 |  |  |
| Frontal_Sup_2_R | [26 14 56] | 5.15 | 0.126 | 93 |  |

| Non-responders > Responders |  |  | peak-level | | cluster-level | |
| --- | --- | --- | --- | --- | --- | --- |
| Region | MNI-Coordinates | T | P _FWE-corr_ | | k_E_ |  |
| Temporal_Mid_L | [-54 -62 10] | 4.33 | 0.993 | 107 | |  |
|  |  |  |  |  | |  |

Table S5: Seed-to-voxel resting-state functional connectivity differences between responders and non-responders across all sessions. Models controlled for age, sex, depression, and anxiety scores. Results reported at uncorrected voxel-wise threshold *p* < 0.001 with cluster-level FWE correction (cluster extent = 93 voxels). No clusters survived voxel-wise FWE correction (*p* < 0.05).

## Whole-brain analysis of the neurofeedback training task

|  |  |  | peak-level | cluster-level |
| --- | --- | --- | --- | --- |
| Region | MNI-Coordinates | T | P _FWE-corr_ | k_E_ |
| Cerebelum_6_L | [-8 -64 14] | 31.52 | <0.001 | 34514 |
| Frontal_Sup_2_L | [-24 -4 62] | 21.11 |  |  |
| Thal_VL_R | [16 -6 6] | 10.84 |  |  |
| Frontal_Mid_2_R | [36 44 32] | 15.34 | <0.001 | 329 |
| Frontal_Mid_2_R | [40 40 24] | 8.2 |  |  |
| Frontal_Mid_2_R | [36 36 40] | 7.84 |  |  |
| Parietal_Inf_R | [52 -38 52] | 12.76 | <0.001 | 737 |
| SupraMarginal_R | [50 -32 38] | 11.41 |  |  |
| Postcentral_R | [56 -16 42] | 10.47 |  |  |
| Precentral_L | [-50 0 40] | 10.47 | <0.001 | 210 |
| Precental_L | [-52 8 40] | 9.8 |  |  |
| Frontal_Inf_Oper_L | [-56 8 24] | 7.44 |  |  |
| Rolandic_Oper_L | [-38 -30 20] | 9.00 | 0.001 | 37 |
| Frontal_Mid_2_L | [-32 38 36] | 8.68 | <0.001 | 75 |
| Frontal_Mid_2_L | [-32 30 34] | 8.16 |  |  |
| Postcentral_L | [-58 -18 20] | 7.87 | 0.001 | 39 |

Table S6: Whole-brain group-level neurofeedback activation results for the contrast *upregulation* > *baseline* across all runs. The table lists the regions that showed significant activation during neurofeedback training across all nine runs (*n* = 18). The results are thresholded at *T* = 6 (uncorrected at the voxel level, *p* <0.001), with a cluster-level family-wise error (FWE) correction applied (*p* < 0.05, FWE_c_ = 39). Significant clusters were observed in regions including the cerebellum, the left superior frontal gyrus, the right middle frontal gyrus, and the right inferior parietal cortex. Sub-clusters within these regions survived voxel-wise FWE correction (*p* < 0.05), indicating robust localized activation. Regions are labelled according to the AAL3 atlas (Rolls et al., 2020). k_E_ = cluster extent.

##

## Baseline comparison of responders and non-responders

|  |
| --- |

| Characteristic | Responders (n = 8) | Non-responders (n =10) | Statistics |
| --- | --- | --- | --- |
| Age, years | 39.6 (8.79) | 36 (11.8) | ns |
| Sex, female / male | 5 / 3 | 8 / 2 | ns |
| Handedness, right / left /ambidextrous | 8 / 0 / 0 | 8 / 1 / 1 | ns |
| Symptom type | 4 weakness, 3 sensorimotor, 5 gait disorder, 2 myoclonus, 3 seizures, 1 dystonia, | 7 weakness, 5 sensorimotor, 2 gait disorder, 3 myoclonus, 1 seizure, 2 tremor, 1 speech disorder | ns |
| ICD-10 classification | 5 mixed FND (F44.7), 2 motor FND (F44.4),  1 functional seizure (F44.5) | 6 mixed FND (F44.7), 3 motor FND (F44.4),  1 functional seizure (F44.5) | ns |
| Psychotropic medication | 4 none  4 antidepressants | 4 none  1 benzodiazepine  4 antidepressants  2 neuroleptics  2 antiepileptics  2 opioids | ns |
| Duration of illness, years | 4.8 (4.3) | 6.3 (4.2) | ns |
| Disease severity, CGI | 3.4 (1.2) | 2.9 (1.1) | ns |
| Disease severity, S-FMDRS | 9.8 (7.6) | 6.6 (4.6) | ns |
| SSS | 40.5(26.7) | 56.1(22.3) | ns |
| BDI | 18 (11.8) | 14.2(9.8) | ns |
| STAI |  |  |  |
| Y1 (state) | 40.6 (12.7) | 33 (12.5) | ns |
| Y2 (trait) | 46.8 (9.9) | 42.4 (15.5) | ns |
| Neuro-QoL  Social Roles  Satisfaction Social Roles  Cognition Function | 25.1(8.1)  25.5(8.8)  22(8.7) | 22.9(5.9)  21.1(5.7)  23(7.3) | ns  ns  ns |
| SF-36  Physical Component  Mental Component | 34.27(12.59)  44.24(10.51) | 42.2(10.65)  38.2(9.58) | ns  ns |

Table S7: Baseline group comparisons between responders and non-responders. Group comparisons were conducted using Fisher’s exact test for categorical variables, and independent *t*-tests or Mann–Whitney U tests for continuous variables, depending on the distribution. Questionnaire subscales were adjusted for multiple comparisons using false discovery rate correction (*q*_FDR_ = 0.05). “ns” indicates non-significant differences between groups.

## Cognitive strategies underlying neurofeedback training

To explore whether individual differences in cognitive strategies contributed to variability in neurofeedback response, we analyzed participant-reported strategies across the three NF training sessions (Fig. S6). This analysis was exploratory and descriptive, aiming to identify potential trends rather than test specific hypotheses.

Participants most commonly reported task-related strategies such as focusing attention on the game mechanics or anticipating target locations. Non-task-related strategies included relaxation techniques (e.g., mindfulness) or disengaging by thinking about unrelated topics. While both groups employed a range of strategies, responders tended to report task-focused approaches more frequently, whereas non-responders more often described strategies reflecting disengagement from the task. However, no significant association was found between subgroups (responders vs. non-responders) and the type of cognitive strategy used (*p* = 0.53, two-sided Fisher’s exact test).

The use of strategies over time showed a similar descriptive pattern (Fig. S7): responders maintained a high proportion of task-related strategies (approximately 80% of all strategies reported within the group) across all sessions. In contrast, non-responders showed a decrease from approximately 75% in session 1 to below 50% in session 3, suggesting a gradual shift toward more non-task-related strategies. This pattern may indicate a decline in cognitive engagement with the task over time.


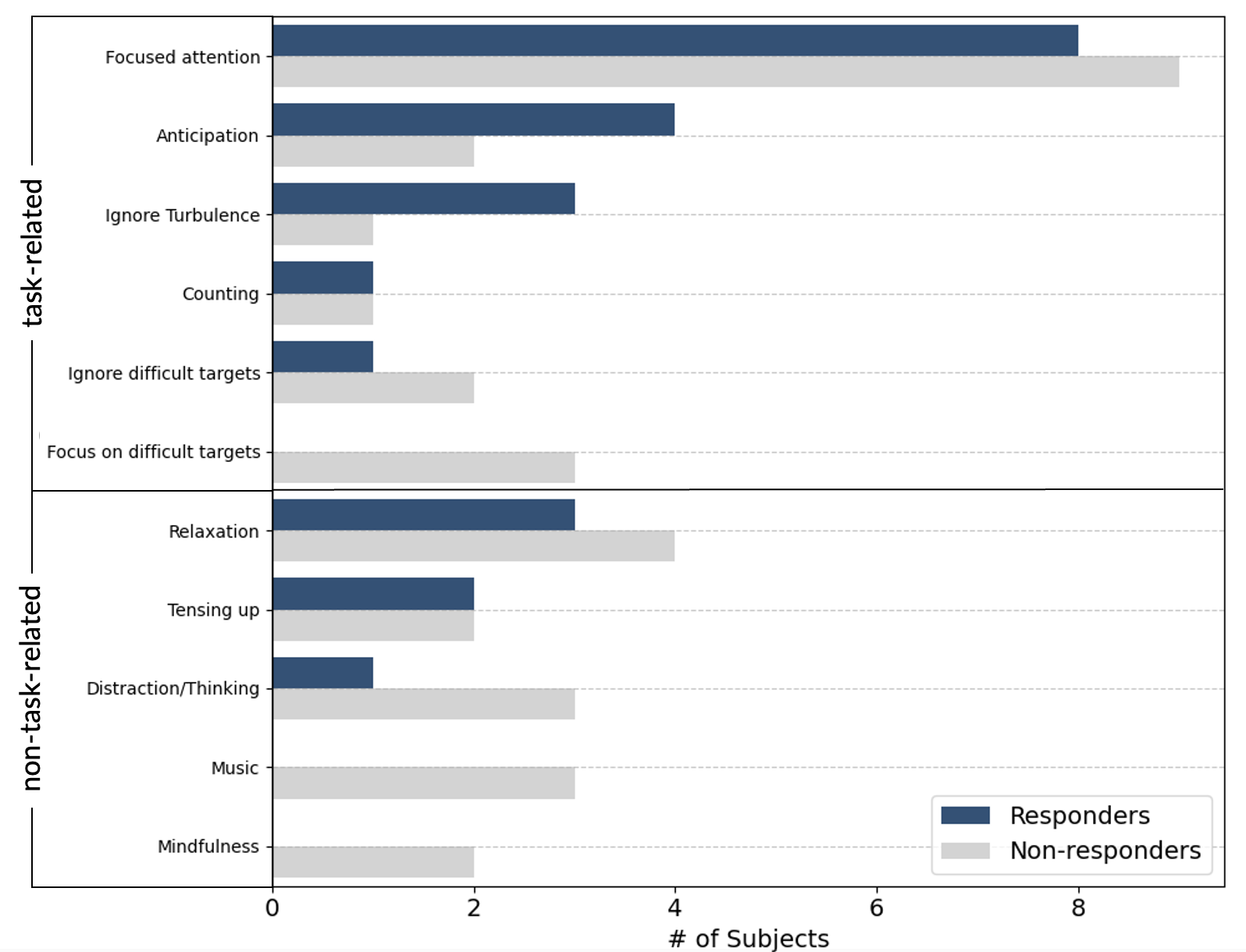


Fig. S6: Self-reported strategies used during neurofeedback training in responders and non-responders. An extended Fisher's exact test revealed no significant association between groups (responders vs. non-responders) and reported strategy use. Task-related strategies are depicted at the top of the graph, while non-task-related strategies are shown at the bottom. Participants reported multiple strategies.


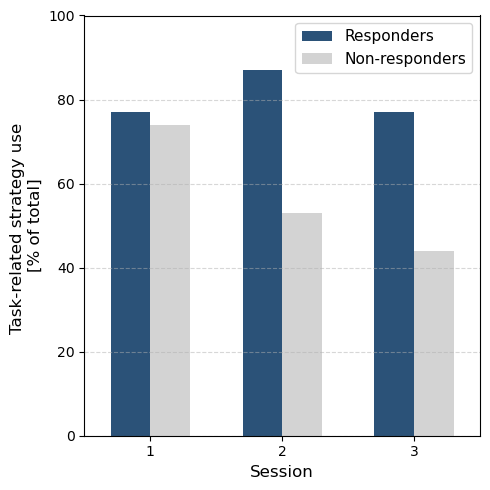


Fig. S7: Task-related strategy use across sessions in responders and non-responders. Bars show the percentage of reported strategies classified as task-related among all strategies used in each session within the group. Responders maintained consistently high levels of task-related strategy use, while non-responders showed a progressive decline across sessions.

## Task performance during the agency task
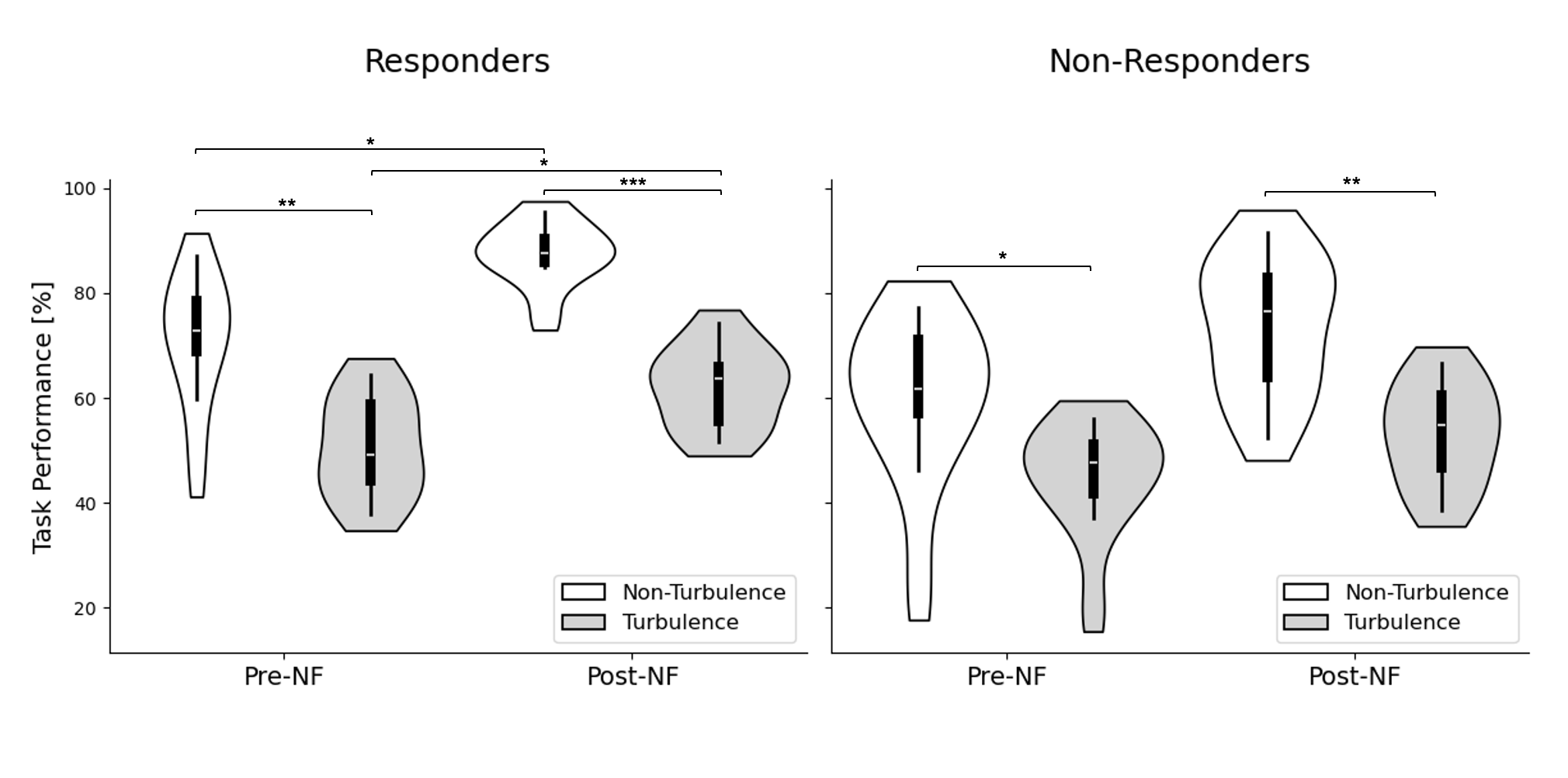


##

Fig. S8: Changes in task performance during the agency task for responders and non-responders. Performance was defined as the difference between the percentage of targets hit and the percentage of distractors hit, with higher values indicating better discrimination between relevant and irrelevant stimuli. Group mean contrast estimates are shown across sessions for both the *turbulence* and *non-turbulence* conditions. Responders showed a significant improvement in task performance over time in both conditions, indicating a learning effect. In contrast, non-responders did not show significant changes in performance across sessions. At baseline (i.e., the first agency task session), there were no significant differences in task performance between responders and non-responders. * *p* < 0.05, ** *p* < 0.01, *** *p* < 0.001.

## Task performance during the neurofeedback training task
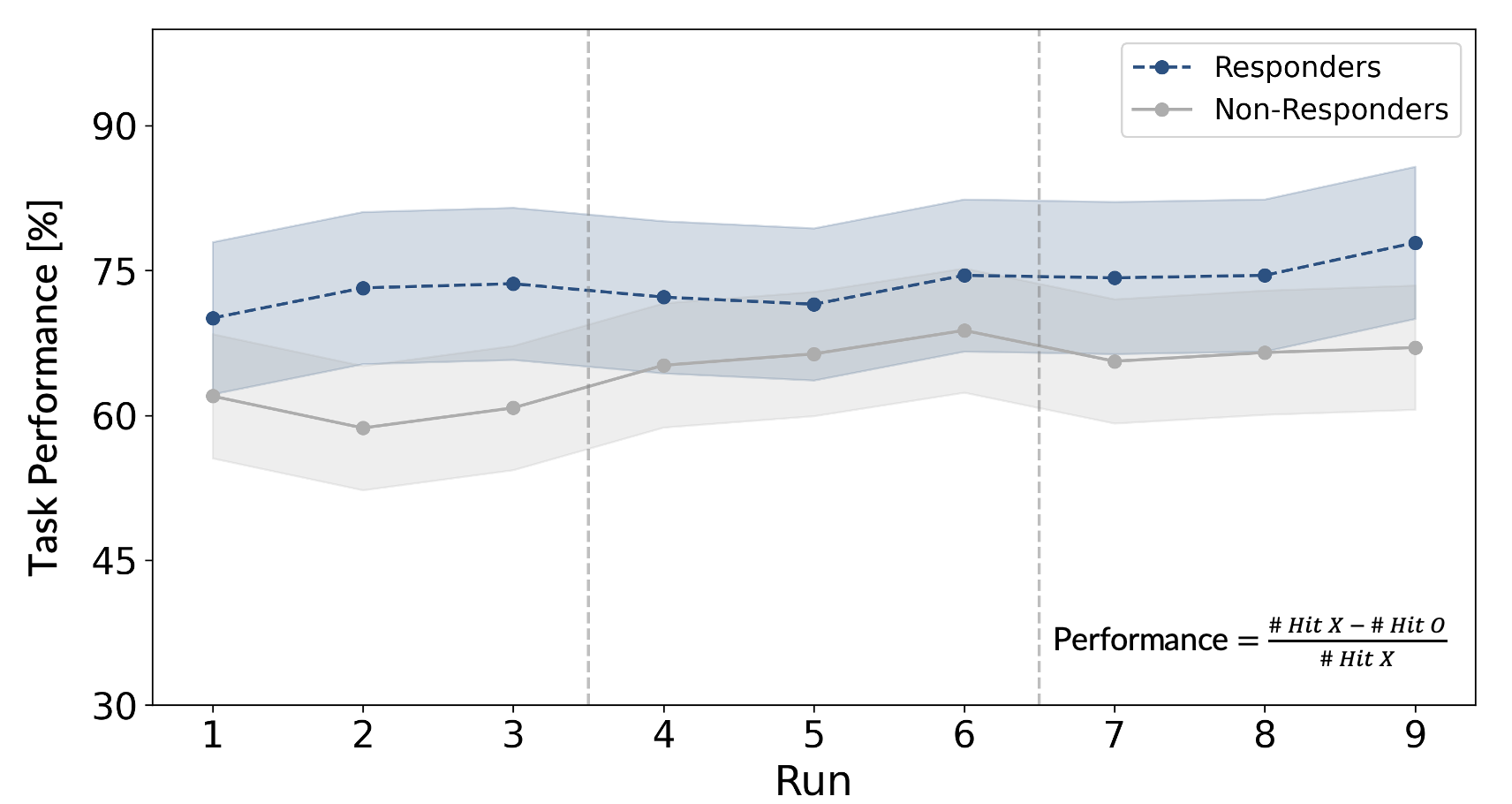


Fig. S9: Changes in task performance during neurofeedback training for responders and non-responders*.* Task performance was defined as the difference between the percentage of targets hit and the percentage of distractors hit. Group mean contrast estimates per run are shown with 95% confidence intervals, based on model-fitted data after adjustment for covariates (age, sex, depression, and anxiety scores). A linear mixed-effects model was fitted, followed by a mixed ANOVA on the model's output. The results showed a significant main effect of run (*F* = 3.55, *p* = 0.0009) and a trend-level main effect of group (*F* = 4.48, *p* = 0.054). The interaction between run and group was not significant (*F* = 1.63, *p* = 0.12), indicating that performance changed over time but did not differ significantly between groups in terms of slope.

## Behavioral-neuroimaging associations

| JoA vs. | rTPJ | lTPJ | SMA | IC | dlPFC |
| --- | --- | --- | --- | --- | --- |
| Agency task  ROI activity | .07 [-.54,.53] | -.18 [-.6, .46] | -.21 [-.67, .31] | .01 [-.51, .53] | -.03 [-.56, .49] |
| Agency task  FC | N.A. | -.11 [-.56, .44] | .55 [-.89, -.09] | -.06 [-.63, .49] | -.45 [-.88, .05] |
| NF training  ROI activity | -.55 [-.88, -.13] | -.31 [-.72, .21] | -.51 [-.86, .29] | -.54 [-.88, -.27] | -.45 [-.78, -.01] |

Table S8: Associations between JoA change (post–pre-NF) and neuroimaging metrics. Neuroimaging metrics included (i) changes in agency task ROI activity (post–pre-NF), (ii) changes in agency task functional connectivity (post–pre-NF), and (iii) changes in ROI activity during NF training (last–first session). Values show Spearman’s ⍴ with 95% bias-corrected and accelerated (BCa) confidence intervals based on 10,000 bootstrap samples, adjusted for age, sex, and depression/anxiety scores. Correlations significant at *p* < 0.05 (uncorrected) are in bold; none survived FDR correction. FC, functional connectivity. N.A., not applicable, ROI, region of interest.

## Clinical-neuroimaging associations

|  | rTPJ | lTPJ | SMA | IC | dlPFC |
| --- | --- | --- | --- | --- | --- |
| SFMDRS | -.05 [-.45, .49] | .06 [-.43, .55] | -.18 [-.61, .34] | -.26 [-.71, .24] | .05 [-.4, .59] |
| CGI | -.51[-.83, -.08] | -.31 [-.78, .05] | -.48 [-.74, .08] | -.20 [-.70, .15] | -.50 [-.84, -.04] |
| Illness duration | .30 [-.31, .72] | .15[-.38, .62] | .44 [-.22, .74] | .25 [-.35, .65] | .05 [-.54, .46] |
| SSS | .06 [-.57, .47] | .11[-.43, .64] | .07 [-.60, .54] | .33 [-.17, .71] | -.15 [-.64, .32] |
| NQ Social | -.04 [-.50, .55] | .08 [-.25, .72] | .13 [-.43, .67] | .01 [-.41, .61] | -.07 [-.47, .55] |
| NQ Cognition | -.31 [-.76, .10] | -.27 [-.68, .30] | -.27 [-.69, .23] | .16 [-.46, .68] | -.36 [-.8, .03] |
| NQ Satisfaction | .36 [-.17, .73] | .50 [.09, .78] | .49 [-.12, .80] | .30 [-.31, .67] | .22 [-.25, .64] |
| SF Physical comp | -.25 [-.25, .58] | -.26 [-.19, .70] | -.38 [-.04, .89] | -.31 [-.12, .80] | -.32 [-.30, .68] |
| SF Mental comp | .27 [-.21, .68] | .25 [-.18, .73] | .42 [-.04, .79] | .49 [.04, .80] | .27 [-.42, .68] |

Agency task ROI activity vs. clinical outcomes

Table S9: Associations between ROI activation during the agency task and changes in clinical outcomes. Spearman rank correlations between changes in volitional control (post–pre-NF ROI activity during the agency task) and changes in clinical variables (post–pre-NF). Values reflect Spearman’s *⍴* and 95% bias-corrected and accelerated (BCa) confidence intervals based on 10,000 bootstrap samples (corrected for the covariates age, sex, and depression/anxiety scores). None of the associations was significant.

Agency task functional connectivity vs. clinical outcomes

|  | lTPJ | SMA | IC | dlPFC |
| --- | --- | --- | --- | --- |
| SFMDRS | -.33 [-.61, .45] | -.41 [ .75, .13] | -.41 [-.77, .19] | -.17 [-.69, .41] |
| CGI | -.12 [-.59,.45] | -.28 [-.62, .44] | -.29 [-.59, .35] | -.08 [-.45, .50] |
| Illness duration | .21 [-.19, .80] | .03 [-.34, .69] | .17 [-.36, .53] | -.04 [-.44, .47] |
| SSS | .02 [-.53, .48] | -.06 [-.65, .30] | -.20 [-.60, .35] | -.21 [-.64, .41] |
| NQ Social | .04 [-.44, .53] | -.41 [-.75, .28] | .06 [-.47, .53] | .38 [-.15, .78] |
| NQ Cognition | -.04 [-.52, .37] | -.18 [-.56, .27] | -.10 [-.57, .38] | -.05 [-.46, .56] |
| NQ Satisfaction | .02 [-.41, .65] | -.57 [-.62, .28] | .09 [-.39, .62] | .33 [-.24, .72] |
| SF Physical comp | .36 [-.23, .66] | .36 [-.28, .71] | -.17 [-.65, .36] | -.24 [-.71, .29] |
| SF Mental comp | .36 [-.37, .68] | .36 [-.19, .72] | .62 [.06, .85] | -.24 [-.25, .74] |

Table S10: Associations between task-based functional connectivity and clinical outcomes. Spearman correlations between changes in task-related functional connectivity and changes in clinical measures. Results show Spearman’s ⍴ with 95% BCa confidence intervals (10,000 bootstrap samples) adjusted for age, sex, and depression/anxiety scores. Values in bold indicate correlations significant at *p* < 0.05 (uncorrected for multiple comparisons). No values survived FDR correction for multiple comparisons.

NF training ROI activity vs. clinical outcomes

|  | rTPJ | lTPJ | SMA | IC | dlPFC |
| --- | --- | --- | --- | --- | --- |
| SFMDRS | -.01 [-.44, .57] | .13 [-.30, .73] | .04 [-.31, .58] | .19 [-.21, .57] | -.01 [-.23, .68] |
| CGI | .17 [-.26, .73] | .2 [-.22, .75] | .11 [-.33, .77] | .35 [.08, .87] | .07 [-.17, .83] |
| Illness duration | .45 [-.30, .66] | .61 [-.20, .63] | .52 [-.37, .63] | .16 [-.55, .48] | .38 [-.38, .54] |
| SSS | -.78 [-.86, -.38] | -.55 [-.74, .14] | -.48 [-.80, -.04] | -.63 [-.82, -.34] | -.75 [-.84, -.06] |
| NQ Social | .15 [-.28, .63] | .26 [-.12, .68] | .35 [-.10, .60] | .27 [-.32, .60] | .15 [-.25, .70] |
| NQ Cognition | -.04 [-.51, .46] | .06 [-.51, .45] | .03 [-.5, .54] | -.07 [-.57, .57] | -.17 [-.58, .39] |
| NQ Satisfaction | -.08 [-.58, .31] | .14 [-.50, .41] | .06 [-.53, .41] | -.15 [-.53, .41] | -.03 [-.58, .42] |
| SF Physical Comp | -.46 [-.74, -.01] | -.55 [-.74, -.15] | -.46 [-.7, -.15] | -.09 [-.60, .29] | -.45 [-.78, -.02] |
| SF Mental Comp | .41 [-.14, .72] | .33 [-.32, .60] | .43 [-.24, .68] | -.05 [-.52, .46] | .31 [-.43, .63] |

Table S11: Associations between changes in neurofeedback-related activation and clinical outcome measures. Spearman correlations between changes in ROI activation during neurofeedback training (last session - first session) and clinical variables (post – pre-NF training). Values reflect Spearman’s *⍴* and 95% bias-corrected and accelerated (BCa) confidence intervals based on 10,000 bootstrap samples. Values in blue indicate correlations that survived FDR correction (*q*_FDR_ = 0.05). Values in bold indicate significance at *p* < 0.05, which did not survive FDR correction.

References

Kasper, L., Bollmann, S., Diaconescu, A.O., Hutton, C., Heinzle, J., Iglesias, S., Hauser, T.U., Sebold, M., Manjaly, Z.-M., Pruessmann, K.P., Stephan, K.E., 2017. The PhysIO Toolbox for Modeling Physiological Noise in fMRI Data. Journal of Neuroscience Methods 276, 56–72. https://doi.org/10.1016/j.jneumeth.2016.10.019

Kleiner, M., Brainard, D., Pelli, D., Ingling, A., Murray, R., Broussard, C., 2007. What’s new in psychtoolbox-3. Perception 36, 1–16.

Lakens, D., 2013. Calculating and reporting effect sizes to facilitate cumulative science: a practical primer for t-tests and ANOVAs. Front. Psychol. 4. https://doi.org/10.3389/fpsyg.2013.00863

Rolls, E.T., Huang, C.-C., Lin, C.-P., Feng, J., Joliot, M., 2020. Automated anatomical labelling atlas 3. NeuroImage 206, 116189. https://doi.org/10.1016/j.neuroimage.2019.116189

Smith, S.M., 2002. Fast robust automated brain extraction. Hum Brain Mapp 17, 143–155. https://doi.org/10.1002/hbm.10062

Whitfield-Gabrieli, S., Nieto-Castanon, A., 2012. Conn: a functional connectivity toolbox for correlated and anticorrelated brain networks. Brain Connect 2, 125–141. https://doi.org/10.1089/brain.2012.0073
